# Quantitative Acetylomics Revealed Acetylation-Mediated Molecular Pathway Network Changes in Human Nonfunctional Pituitary Neuroendocrine Tumors

**DOI:** 10.3389/fendo.2021.753606

**Published:** 2021-10-12

**Authors:** Siqi Wen, Jiajia Li, Jingru Yang, Biao Li, Na Li, Xianquan Zhan

**Affiliations:** ^1^ Key Laboratory of Cancer Proteomics of Chinese Ministry of Health, Central South University, Changsha, China; ^2^ Medical Science and Technology Innovation Center, Shandong First Medical University, Jinan, China; ^3^ Shandong Key Laboratory of Radiation Oncology, Shandong Cancer Hospital and Institute, Shandong First Medical University, Jinan, China; ^4^ Gastroenterology Research Institute and Clinical Center, Shandong First Medical University, Jinan, China

**Keywords:** acetylomics, label-free quantitative proteomics, gene ontology (GO), signaling pathway, biomarker, pituitary neuroendocrine tumor (PitNET)

## Abstract

Acetylation at lysine residue in a protein mediates multiple cellular biological processes, including tumorigenesis. This study aimed to investigate the acetylated protein profile alterations and acetylation-mediated molecular pathway changes in human nonfunctional pituitary neuroendocrine tumors (NF-PitNETs). The anti-acetyl antibody-based label-free quantitative proteomics was used to analyze the acetylomes between NF-PitNETs (n = 4) and control pituitaries (n = 4). A total of 296 acetylated proteins with 517 acetylation sites was identified, and the majority of which were significantly down-acetylated in NF-PitNETs (p<0.05 or only be quantified in NF-PitNETs/controls). These acetylated proteins widely functioned in cellular biological processes and signaling pathways, including metabolism, translation, cell adhesion, and oxidative stress. The randomly selected acetylated phosphoglycerate kinase 1 (PGK1), which is involved in glycolysis and amino acid biosynthesis, was further confirmed with immunoprecipitation and western blot in NF-PitNETs and control pituitaries. Among these acetylated proteins, 15 lysine residues within 14 proteins were down-acetylated and simultaneously up-ubiquitinated in NF-PitNETs to demonstrate a direct competition relationship between acetylation and ubiquitination. Moreover, the potential effect of protein acetylation alterations on NF-PitNETs invasiveness was investigated. Overlapping analysis between acetylomics data in NF-PitNETs and transcriptomics data in invasive NF-PitNETs identified 26 overlapped molecules. These overlapped molecules were mainly involved in metabolism-associated pathways, which means that acetylation-mediated metabolic reprogramming might be the molecular mechanism to affect NF-PitNET invasiveness. This study provided the first acetylomic profiling and acetylation-mediated molecular pathways in human NF-PitNETs, and offered new clues to elucidate the biological functions of protein acetylation in NF-PitNETs and discover novel biomarkers for early diagnosis and targeted therapy of NF-PitNETs.

## Introduction

Pituitary neuroendocrine tumors (PitNETs) are the second most common primary central nervous system tumors in adults (1, 2). Based on serum hormone level, PitNETs are divided into clinically functional and nonfunctional PitNETs (F-PitNETs and NF-PitNETs). NF-PitNETs account for 15% to 54% of diagnosed PitNETs (3). F-PitNETs patients are generally diagnosed at early stage because of their hormonal hypersecretory syndrome and some of them are obtained efficient medical therapies to inhibit pituitary hormone secretion. Whereas, NF-PitNETs are not easily diagnosed at early stage because of no hormonal hypersecretory syndrome and lack effective medicine for noninvasive therapy (4). Currently, the only way to control NF-PitNET tumor mass effect (hypophysis dysfunction, visual field defect, and headache) is surgical resection. However, some NF-PitNETs seem to be more invasive and have higher postoperative recurrence rates, which severely decrease the quality of life of patients (5, 6). NF-PitNETs are becoming a challenging clinical problem. It is necessary to clarify molecular mechanisms of the occurrence and development of NF-PitNETs, and discover effective biomarkers for early diagnosis and treatment of NF-PitNETs to improve their quality of life.

Acetylation is a reversible post-translational modification (PTM), and is co-regulated by lysine acetyltransferases (KATs) and lysine deacetylases (KDACs). KATs catalyze lysine residue to be acetylated with acetyl-coenzyme A (acetyl-CoA) as a cofactor, and KDACs reverse this process. Acetylation modulates biological functions of many proteins related to tumorigenesis. Histone acetylation facilitates chromatin decondensation to regulate transcriptional activation (7). When DNA suffers from damage, particular sites of p53 protein are acetylated to modulate its functions in damage repair or cell apoptosis (8). The level of c-MYC oncoprotein tightly relates to cell cycle progression, its acetylation dramatically increases its protein stability (9). Study found that many cancers existed aberrant expression, mutation, and translocation of one of specific lysine acetylation regulators (KATs, KDACs, and acetyl-lysine readers) or a group of them (10). These abnormalities might impact stabilities and expressions of many oncoproteins, tumor suppressor proteins, chaperones, and other functional proteins by altering their acetylation levels to initiate some of cancer-related signaling pathways and affect tumor growth, invasion, and metastasis (8, 9, 11–16). Thereby, it emphasizes that the altered acetylation levels of proteins have potential to affect tumorigenesis and development of NF-PitNETs.

However, acetylomics analysis in NF-PitNETs has not been reported. Previous studies on the effect of acetylation on pituitary tumorigenesis only focused on some specific molecules (17–19). Thus, elucidation of acetylome in NF-PitNETs might offer new insights into the role of lysine acetylation played in the pathophysiology of NF-PitNETs and lead to the discovery of novel biomarkers for its early diagnosis and efficacious therapeutic targets.

Anti-acetyl antibody-based label-free quantitative proteomics is widely used to detect, identify, and quantify acetylome in a given condition, such as substrates of acetyltransferases and deacetylases, and tumors *vs.* controls (16, 20, 21). This study selected a rigorous anti-acetyl antibody-based label-free quantitative mass spectrometry (MS) to identify and quantify acetylated proteins between NF-PitNET and control pituitary tissues. Subsequently, functional and pathway network analyses were performed to investigate the functional characteristics of differentially acetylated proteins (DAPs) and molecular network alterations that protein acetylation was involved in. The randomly selected acetylation status of phosphoglycerate kinase 1 (PGK1) that was identified with acetylomics in NF-PitNETs relative to normal pituitaries was confirmed with immunoprecipitation and western blot analyses.

In addition, approximately one-third of acetylation sites are also subjected to ubiquitination in human cells, which presents a competition and synergy relationship between acetylation and ubiquitination (22). Some proteins involved in important biological processes might affect tumor formation and progression through the regulatory crosstalk between acetylation and ubiquitination, such as p53, histone H3, and splicing factor SRSF5 (23–25). Thereby, an overlapping analysis between acetylated proteins data and ubiquitinated proteins data identified from the same NF-PitNET and control pituitary samples was performed to investigate the potential competition and synergy effects of protein acetylation and ubiquitination on NF-PitNETs.

Moreover, invasiveness is a challenging clinical problem. This study further investigated the relationship of protein acetylation and invasive characteristics in NF-PitNETs. Differentially expressed genes (DEGs) were obtained between invasive NF-PitNETs and control tissues from Gene Expression Omnibus (GEO) database. The overlapping analysis between acetylated protein data and invasive DEG data was performed to identify acetylation-mediated molecular events for invasiveness of NF-PitNETs.

This study will provide promising scientific data for insights into molecular mechanisms of NF-PitNETs, and discover potential biomarkers for early diagnosis and therapy of NF-PitNET patients.

## Materials and Methods

### Tissue Samples

Quantitative acetylomics was performed between the mixed NF-PitNET samples (n =4) and mixed control samples (n =4) (**Supplementary Table 1**). NF-PitNET samples were obtained from Department of Neurosurgery, Xiangya Hospital, China, with approval of the Xiangya Hospital Medical Ethics Committee of Central South University. Control pituitary tissues were obtained from the Memphis Regional Medical Center, with approval of the University of Tennessee Health Science Center Internal Review Board. Each sample was collected after obtaining written informed consent from each patient or the family of each control pituitary subject (autopsy tissues). The detailed information on samples was described previously (26), and collected (**Supplementary Table 1**).

### Protein Extraction and Quality Assessment of Protein Sample

Each tissue sample was dealt with 1 mL urea pyrolysis solution [9 M urea (U5378, Merck), 20 mM 2-hydroxyethyl (HEPES; H3375, Merck), 1 mM sodium orthovanadate (S6508, Merck), 2.5 mM sodium pyrophosphate (P8010, Merck), and 1 mM β-glycerophosphate (G9422, Merck), pH 8.0] and ice bath ultrasonic treatment. The solution was centrifuged (18000×g, 30 min, and 4°C), and the supernatant of each sample was equally divided into three parts. The protein content of each part was measured with a Bradford Protein Quantification Kit (YEASEN, Cat# 20202ES76). An amount (20 µg) of each extracted protein sample (NF-PitNETs; Controls) was mixed with 6X loading buffer (P0015F, Boyetime) in a ratio of 6:1(v/v), boiled (5 min), and centrifuged (14000×g, and 10 min). The supernatant was loaded onto 12.5% SDS-PAGE gel (P0012A, Boyetime) for electrophoretic separation (constant current 15 mA, and 60 min), followed by staining with Coommassie brilliant blue (P0017A, Boyetime).

### Enzymatic Hydrolysis of Proteins

Dithiothreitol (DTT; D9760, Merck) was added to each extracted protein sample (NF-PitNETs; Controls), and achieved a final concentration of 10 mM DTT; and the mixture was incubated (2.5h, and 37°C), and cooled to room temperature. Then indole-3-acetic acid (IAA; I3750, Merck) was added into each mixture, and achieved a final concentration of 50 mM IAA; and the mixture was kept (dark, 30 min). The water that was 5 times the volume of the mixture was added to make the concentration of urea to 1.5 M, followed by addition of trypsin into the mixture in a ratio of 1:50 to digest proteins for 18 h at 37 °C. The SPE C18 column (Waters WAT051910, Waters Corporation, Milford, CT, USA) was used to desalt and lyophilize tryptic peptides.

### Enrichment of Acetylated Peptides

A volume (1.4 mL) of pre-cooled immunoaffinity purification (IAP) buffer was used to resuspend each lyophilized peptide sample (3x). The pre-processed anti-Ac-K antibody beads [PTMScan Acetyl-Lysine Motif (Ac-K) Kit, Cell Signal Technology] were added in each tryptic peptide sample, and incubated (1.5h, 4°C). Afterwards, anti-Ac-K antibody beads with acetylated peptides were washed with 1 mL pre-cooled IAP (3x), and with 1 mL pre-cooled water (3x). A volume (40 μl) of 0.15% trifluoroacetic acid (TFA; 302031, Merck) was added to the washed anti-Ac-K antibody beads, and incubated (room temperature, 10 min), and then the same volume of TFA was added once again. The mixture was centrifuged (2000×g, 30s). The supernatant was desalted with C18 STAGE Tips (27). The desalted supernatant was the enriched acetylated peptide sample.

### LC-MS/MS Analysis of Enriched Acetylated Peptides

LC-MS/MS was used to analyze the enriched acetylated peptides (NF-PitNETs; controls). Each enriched acetylated peptide sample was separated by high performance liquid chromatography (HPLC) system EASY-nLC1000 at nanoliter flow velocity. Chromatography column was balanced with 100% buffer A (0.1% acetonitrile formate aqueous solution that contained 2% acetonitrile). The enriched acetylated peptides were loaded onto the sample spindle, Thermo scientific EASY column (2 cm*100 μm 5 μm-C18), with an autosampler in buffer A, and then were separated when the sample flowed through analytical column (75 µm × 250 mm 3 µm-C18) at a flow rate of 250 nL/min in buffer B (0.1% acetonitrile formate aqueous solution that contained 84% acetonitrile). The liquid-phase gradient was buffer B linear gradient from 0 to 55% for 220 min, buffer B linear gradient from 55 to 100% for 8 min, and then maintained 100% buffer B for 12 min. The Q-Exactive mass spectrometer (Thermo Finnigan) was used to perform MS/MS analysis when the enriched acetylated peptides were separated with capillary HPLC. The parameter of mass spectrometer was set as time 240 min, positive ion detection mode, and scan range of precursor ion *m/z* 350-1800. The top 20 intensive ions in MS scan (MS1) were selected for ion fragmentation with higher-energy collision dissociation (HCD) to generate MS/MS spectra (MS2). The MS1 resolution was 70,000 at *m/z* 200, and the MS2 resolution was 17,500 at *m/z* 200.

### Label-Free Quantification With MaxQuant

The Maxquant software (version 1.3.0.5) was used for database searching and data analysis of 6 original LC-MS/MS datasets (NF-PitNETs: n =3; Controls: n =3). The database was uniprot_human_154578_20160815.fasta (154,578 entries, downloaded on 15 August 2016). Its primary parameters were set as main search ppm = 6, missed cleavage = 4, MS/MS tolerance ppm = 20, de-isotopic =TRUE, enzyme=trypsin, database = uniprot_human_154578_20160815.fasta, fixed modification = carbamidomethyl (C), variable modification = oxidation (M), acetyl (protein N-term), and acetyl (K), decoy database pattern = reverse, iBAQ = TRUE, match between runs = 2 min, peptide false discovery rate (FDR) = 0.01, and protein FDR = 0.01. The MS/MS data were used to determine the amino acid sequence and acetylation sites, label-free quantification was used to determine the acetylation level.

### Immunoaffinity Experiments Confirmed DAPs

Immunoprecipitation (IP) and western blot were used to semi-quantify PGK1 acetylation level in NF-PitNETs compared to controls. Three NF-PitNET tissue samples were equally mixed as the NF-PitNET sample, and five control protein samples were equally mixed as the control sample (**Supplemental Table 1**), which were used to extract protein samples, respectively. An amount (1 mg) of each protein sample (NF-PitNETs; controls) was incubated with the specific antibody against PGK1 (6 μg; sc-130335, Santa Cruz Technology) to immunoprecipitate PGK1 from total proteins. The negative control IP experiment was performed with the use of the normal mouse IgG antibody (6 μg; B900620, Proteintech) to replace the anti-PGK1 antibody, which tested the specificity of anti-PGK1 antibody. The IP products (PGK1 product; IgG product), anti-PGK1 antibody (2 μg), and total protein samples (NF-PitNETs: 60μg; Controls: 60μg) were simultaneously immunoblotted with anti-acetyl-lysine antibody (1:1000; A2391, ABclonal).

### Bioinformatics Analysis of DAPs

DAPs were used for KEGG pathway analysis and gene ontology (GO) analysis through David database. GO analysis included three categories - biological processes (BPs), cellular components (CCs), and molecular functions (MFs). The results of KEGG, BP, CC, and MF data were further clustered into different functional categories. Moreover, acetylation motif analysis was carried out by analysis of the sequences from −13 to +13 amino acid residues at those 517 acetylation sites within 296 acetylated proteins with Motif-X software to identify any motifs that were prone to be acetylated in NF-PitNETs.

### Overlapping Analysis of Acetylated Protein Data and Ubiquitinated Protein Data

The acetylated protein data identified in this study were compared to the ubiquitinated protein data in our previous study (26), which found that acetylation and ubiquitination occurred at the same site in proteins. This overlapping analysis was based on the fact that quantitative acetylomics and quantitative ubiquitinomics were performed in the same samples (NF-PitNETs; controls).

### Overlapping Analysis of Acetylated Protein Data and Invasive DEG Data

In total, 2751 statistically significant DEGs in invasive NF-PitNETs *vs.* controls were mined from the GEO database (**Supplementary Table 2**). The overlapped molecules between 166 DAP data in NF-PitNETs relative to controls and 2751 DEG data in invasive NF-PitNETs relative to controls were obtained, and further analyzed with GO and KEGG pathway enrichments to obtain functional characteristics and signaling pathways mediated by these overlapped molecules.

## Result

### Protein Acetylation Profiling in NF-PitNETs

**Figure 1:**
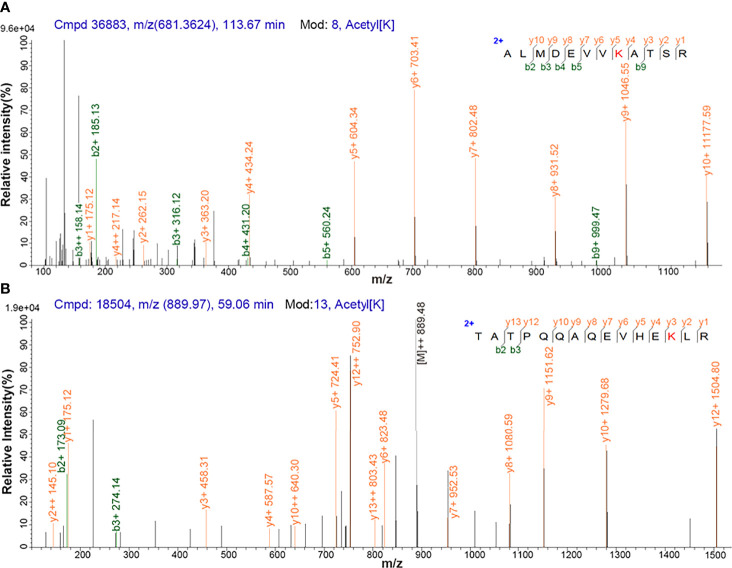
MS/MS spectrum of the tryptic peptide. **(A)** The tryptic peptide ALMDEVVK*ATSR from PGK1. **(B)** The tryptic peptide TATPQQAQEVHEK*LR from triosephosphate isomerase. K* = acetylated lysine residue.

Antibody enrichment-based label-free quantitative acetylomics identified 517 acetylation sites within 296 proteins in NF-PitNETs and control pituitaries (**Supplementary Table 3**). A representative MS/MS spectrum was from acetylated peptide ALMDEVVK*ATSR ([M + 2H]^2+^, *m/z* = 681.4, K* = acetylated lysine residue) derived from PGK1 (Swiss-Prot No.: P00558) (**Figure 1A**), with a high-quality MS/MS spectrum, excellent signal-to-noise (S/N) ratio, and extensive product ions b-ion and y-ion series (b_2_, b_3_, b_4_, b_5_, b_9_, y_1_, y_2_, y_3_, y_4_, y_5_, y_6_, y_7_, y_8_, y_9_, and y_10_). Its acetylation site was localized at residue K*361, which was only acetylated in controls (N) but not in NF-PitNETs (T) (**Supplementary Table 3**). Another representative MS/MS spectrum was from acetylated peptide TATPQQAQEVHEK*LR [(M + 2H)^2+^, *m/z* = 889.5, K* = acetylated lysine residue] of triosephosphate isomerase (Swiss-Prot No.: P60174) (**Figure 1B**), with a high-quality MS/MS spectrum, excellent S/N ratio, and extensive product ions b-ion and y-ion series (b_2_, b_3_, y_1_, y_2_, y_3_, y_4_, y_5_, y_6_, y_7_, y_8_, y_9_, y_10_, y_12_, and y_13_). Its acetylation site was localized at residue K*225, and its acetylation level was significantly decreased in NF-PitNETs compared to controls (ratio of T/N = 0.44; *P* = 3.28E-04) (**Supplementary Table 3**).

**Figure 2:**
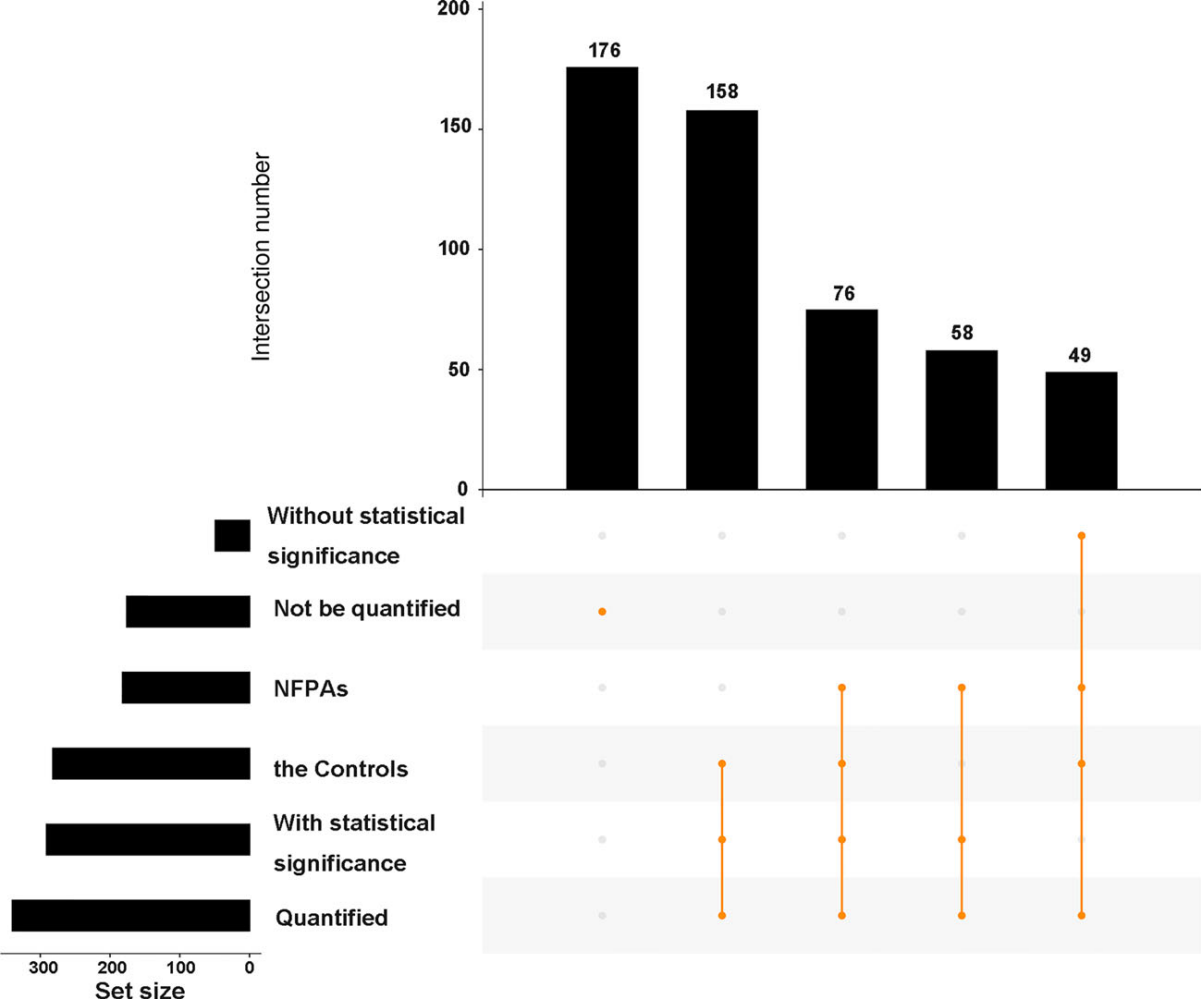
An upset plot showing the overview of 517 identified acetylation sites in 296 proteins in NF-PitNETs relative to controls. All of the identified acetylation sites were classified into 6 groups according to whether quantified or not, whether with statistical significance or not and different sample sources. The horizontal bar chart on the left-hand side that shows the number of acetylation sites identified in NF-PitNET group, control group, statistically significant group, non-statistically significant group, quantified group, and non-quantified group. The intersection matrix in the center of the plot consists of rows that correspond to different groups, and columns that correspond to the intersection sets. The intersections between corresponding groups are presented as vertically connected filled orange circles (exclusive intersections sets). The top vertical bar chart shows the intersection size, the height of which represents the total number of acetylation sites included in the intersection. NFPAs = NF-PitNETs.

**Figure 3:**
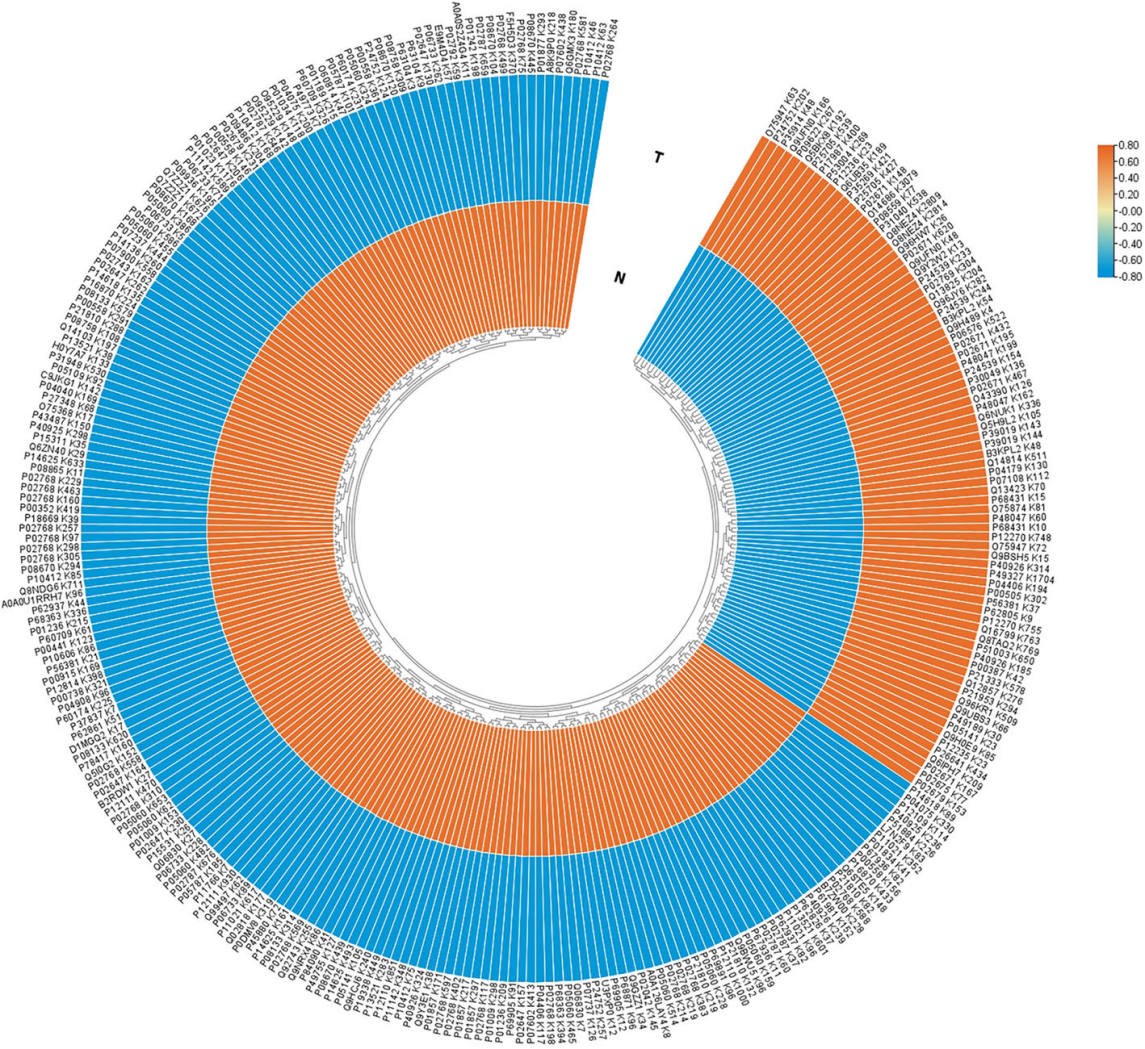
Acetylation quantification heatmap of differentially acetylated lysine sites at proteins in NF-PitNETs and control tissues. “K” means the lysine site. The protein name is able to be got according to the accession number. Orange means increased acetylated level, and blue means decreased acetylated level.

Among these 517 acetylation sites (**Figure 2**), (i) 341 sites were identified and quantified, including 58 sites only quantified in NF-PitNETs, 158 only quantified in controls, and 125 sites quantified in both NF-PitNET and control tissues, and 76 of these 125 sites had statistically significant difference at the acetylation level (p<0.05) in NF-PitNETs compared to controls (53 decreased acetylation levels, and 23 increased acetylation levels); and (ii) 176 sites were only identified but not quantified in neither NF-PitNETs or controls. The acetylation level change of 292 (76 + 58+158) quantified lysine residues with statistically significant difference were visualized by heatmap based on their acetylation intensities in NF-PitNETs and control tissues (**Figure 3**), which revealed that the majority of quantified lysine residues were down-acetylated in NF-PitNETs relative to controls.

### Amino Acid Motifs That Are Prone to Be Acetylated in NF-PitNETs

**Figure 4:**
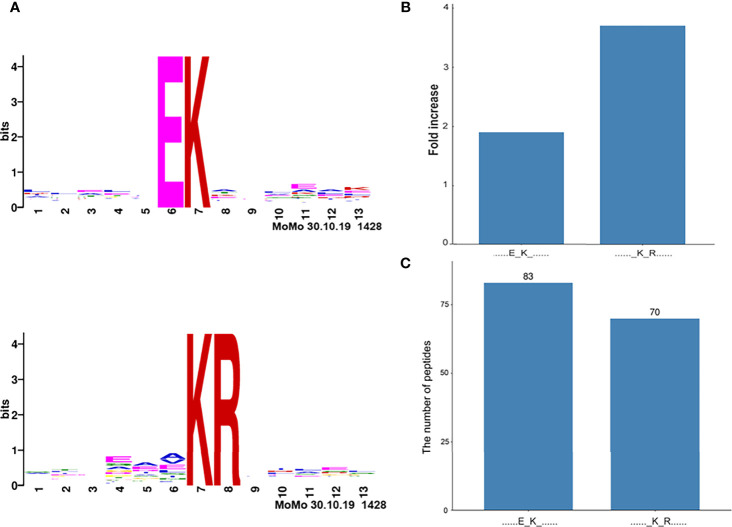
Acetylation motifs of proteins in pituitaries (*Homo sapiens*). **(A)** Two acetylation motifs of proteins predicted with acetylated peptides. The central K stands for the acetylated lysine residue sites in proteins. The size of each letter is related to the frequency of amino acid residues occurring at that position. **(B)** The increased fold-change of each acetylation motif. **(C)** The number of acetylated peptides in each motif. K* = acetylated lysine residue.

Two significantly distinguished motifs EK* and K*R (K* = the acetylated lysine residue) were identified (**Figure 4A**), which referred to 83 and 70 acetylated peptides, respectively. These two types of acetylation motifs had different abundances, which together accounted for 30.5% [(83 + 70)/501] of the identified acetylated peptides (**Figures 4B, C**). It indicates that the residue K within motifs EK and KR is prone to be acetylated in NF-PitNETs.

### Functional Characteristics of DAPs in NF-PitNETs

The functional characteristics of DAPs in NF-PitNETs were analyzed with GO enrichment analysis, including BPs, MFs, and CCs. (i) For BPs, a total of 97 BPs was identified, covering almost all cellular biological processes, including gene expression, metabolism, cell-cell (matrix) adhesion, apoptosis, and immune system regulation (**Supplementary Table 4**). In the aspect of gene expression, those DAPs participated in nucleosome assembly, adenine transport, translation, epigenetics, and post-translational processing and secretion of proteins. In the aspect of metabolism, those DAPs mainly participated in the metabolism of glucose and amino acid, and oxidative phosphorylation to yield ATPs. In the aspect of immune system regulation, those DAPs participated in B cell activation, macrophage phagocytosis, and response to interleukin-4. In addition, DAPs participated in the response to reactive oxygen species, catabolism of superoxide, and affect cellular detoxification process. (ii) For MFs, those DAPs also had extensive MFs (**Supplementary Table 5**). Those DAPs were able to bind mRNA, ADP, NADP, oxygen, cell adhesion molecule, kinds of proteins, enzymes, and receptors, and exert their functions in translation, energy yield, oxygen transport, cell adhesion, proteins processing, cell signal transduction, immune regulation, and catalyzing many kinds of enzyme activities such as oxidoreductase and ubiquitin protein ligase. (iii) For CCs, those DAPs played their roles in different positions. Those DAPs located in almost everywhere in cell, including nucleus, cytoplasm, plasma membrane, and organelles such as mitochondrion, endoplasmic reticulum, and peroxisome. They were also distributed in extracellular region such as cell-cell adherens junction (**Supplementary Table 6**).

**Figure 5:**
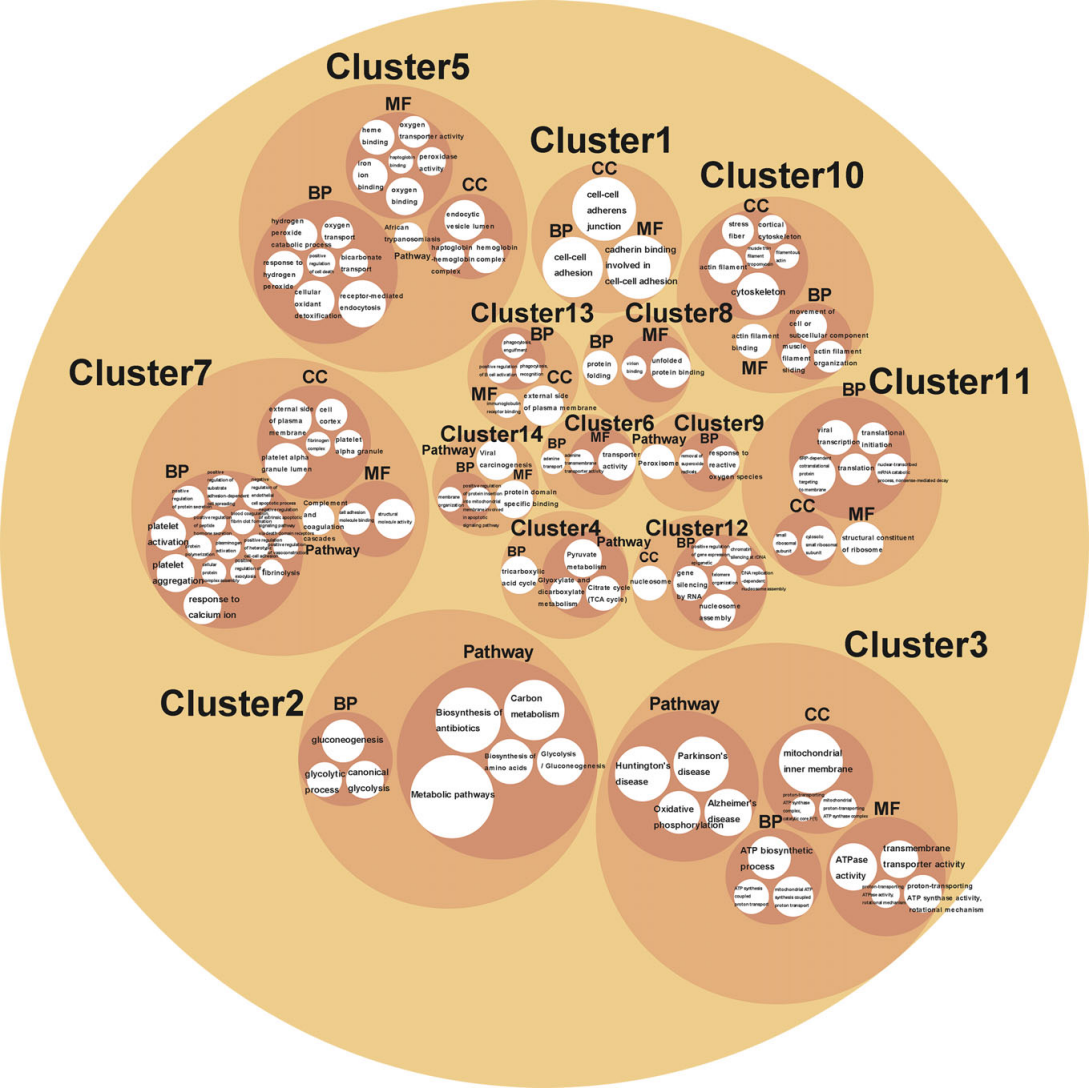
A circle packing chart showing the cluster analysis results. The white circle size is associated to the count of genes enriched on each BP, MF, CC, or pathway. The meaning that each circle represented is annotated in or nearby the circle.

**Figure 6:**
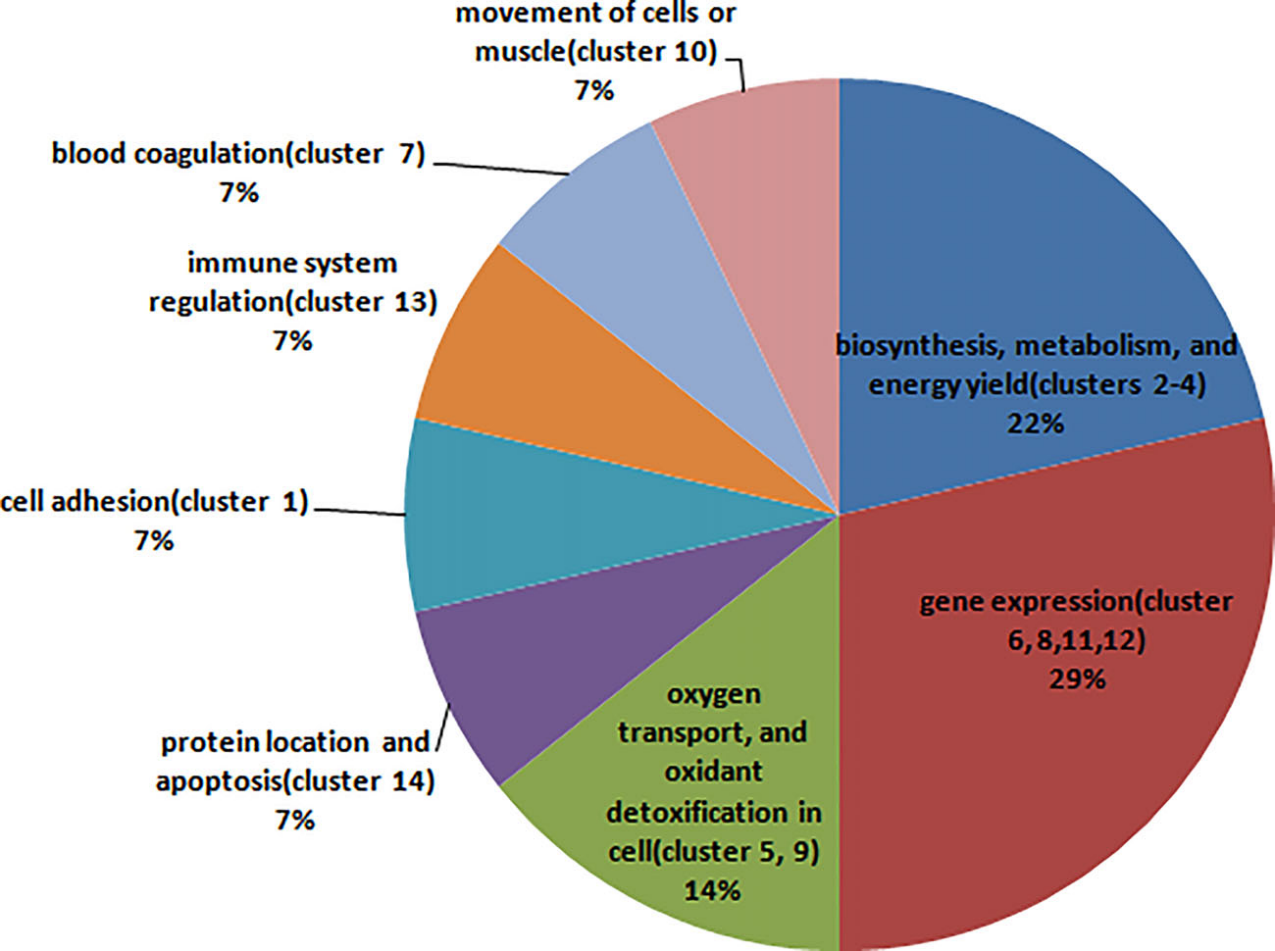
The pie chart shows the main biological function that each cluster involved in, and the percentage that the count of the clusters involved in this biological function accounted for all clusters.

Cluster analysis grouped BPs, MFs, CCs, and KEGG pathways enriched from DAPs into 14 functional clusters (**Figure 5**, **Supplementary Table 7**), including 3 clusters related to biosynthesis, metabolism, and energy yield (Clusters 2-4), 4 clusters related to gene expression (Clusters 6, 8, 11, and 12), 2 clusters related to oxygen transport, and oxidant detoxification in cell (Clusters 5, 9), 1 cluster related to protein location and apoptosis (Cluster 14), 1 cluster related to cell adhesion (Cluster 1), 1 cluster related to immune system regulation (cluster 13), and clusters 7 and 10 related to blood coagulation and movement of cells or muscle, respectively (**Figure 6**).

### Acetylation-Mediated Signaling Pathways in NF-PitNETs

**Figure 7:**
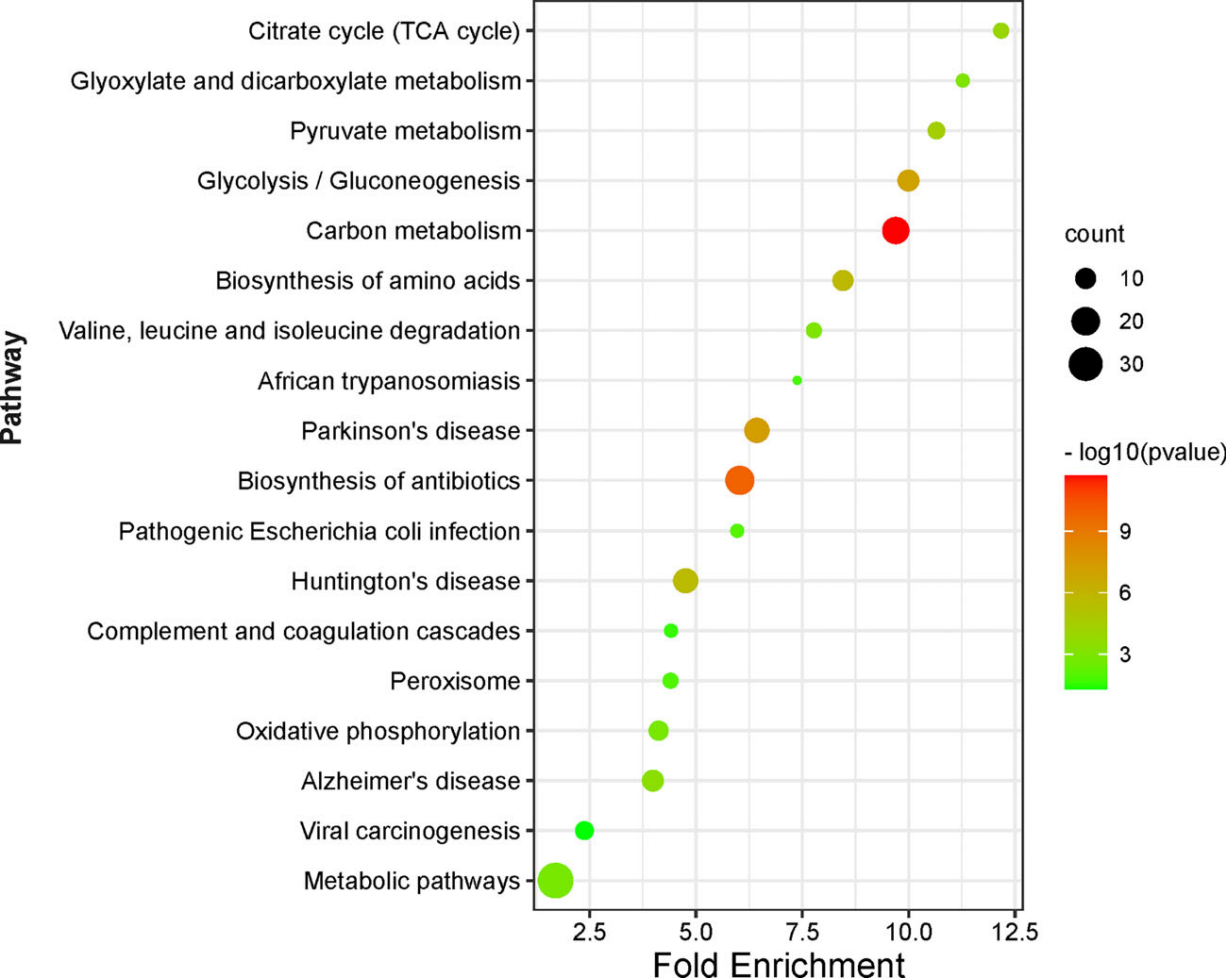
KEGG pathway enrichment analysis of DAPs between NF-PitNETs and the controls. The Y axis shows different pathway terms, the X axis shows fold enrichment. Fold enrichment is calculated as followed: GenehitsGenepathwayHitstotalGenetotal . *Genehits* represents the number of hits in the selected pathway; *Genepathway* represents the number of genes in the selected pathway of KEGG background; *Hitstotal* is the number of total hits in all pathways; *Genetotal* means the number of total genes in all pathways of KEGG background. The circle size represents the count of genes enriched on the pathway. The circle color shows the -log10(p value) of the pathway.

A total 18 statistically significant signaling pathways was identified to involve DAPs with KEGG pathway enrichment analysis (**Figure 7**, **Supplementary Table 8**). Of them, 9 pathways were associated with metabolism and energy yield, 3 associated with nervous system diseases, 3 associated with infectious diseases, 1 was about anti-infection, 1 was about cellular oxidant detoxification, and 1 was about complement and coagulation.

The pathways about metabolism and energy yield included carbon metabolism, glycolysis/gluconeogenesis, pyruvate metabolism, citrate cycle (TCA cycle), glyoxylate and dicarboxylate metabolism, metabolic pathways, biosynthesis of amino acids, oxidative phosphorylation, and valine, leucine and isoleucine degradation. (i) Carbon metabolism consisted of one-carbon metabolism and central carbon metabolism (**Supplementary Figure S1.1**). One-carbon metabolism integrates carbon units from amino acids to generate proteins, nucleotides, and lipids, maintain redox status, and provide substrates for methylation reactions (28). Central carbon metabolism, including glycolysis, TCA cycle, and pentose phosphate pathway, is essential to gain energy from carbohydrate, and provide precursors for many biosynthetic pathways (29). This study found that acetylation mainly occurred at the enzymes that were enriched on the central carbon metabolism, including acetylation at residues K89, K5, K228, K71, and K262 (only acetylated in controls) in alpha-enolase (ID: P06733), K77 (only acetylated in NF-PitNETs) in mitochondrial pyruvate dehydrogenase E1 component subunit alpha (somatic form) (ID: P08559), K135, and K89 (only acetylated in controls) in pyruvate kinase PKM (ID: P14618), K7 (ratio of T/N = 0.58, *p* = 1.52E-02) in transaldolase (ID: P37837), K194 (ratio of T/N =1.74, *p* = 4.24E-03), and K117 (ratio of T/N =0.25, *p* = 2.50E-02) in glyceraldehyde-3-phosphate dehydrogenase (ID: P04406), K81 (ratio of T/N =5.38, *p* = 3.75E-03) in cytoplasmic isocitrate dehydrogenase [NADP] (ID: O75874), K314 (ratio of T/N =2.71, *p*=2.20E-02), K185 (only acetylated in NF-PitNETs), K324 and K239 (only acetylated in controls) in mitochondrial malate dehydrogenase (ID: P40926), K298, and K236 (only acetylated in controls) in cytoplasmic malate dehydrogenase (ID: P40925), K7 (only acetylated in controls) in alcohol dehydrogenase class-3 (ID: P11766), K39 (ratio of T/N =0.06, *p*=7.09E-04) in phosphoglycerate mutase 1 (ID:P18669), K302 (ratio of T/N=1.71, *p*=8.69E-03) in mitochondrial aspartate aminotransferase (ID: P00505), K291, K146, K156 and K361 (only acetylated in controls) in phosphoglycerate kinase 1 (ID: P00558), K538 (only acetylated in NF-PitNETs) mitochondrial succinate dehydrogenase [ubiquinone] flavoprotein subunit (ID: P31040), K231 (only acetylated in controls), and K225 (ratio of T/N=0.44, *p*=3.28E-04) in triosephosphate isomerase (ID: P60174), K169 (only acetylated in controls) in catalase (ID: P04040), K330 and K200 (only acetylated in controls) in fructose-bisphosphate aldolase A (ID: P04075), K202 (ratio of T/N=4.13, *p*=3.34E-02), K257 (ratio of T/N=0.35, *p*=1.13E-02), and K124 (only acetylated in controls) in mitochondrial acetyl-CoA acetyltransferase (ID: P24752), and K267 (ratio of T/N =1.95, *p* =1.42E-04) in mitochondrial dihydrolipoylde hydrogenase (ID: P09622). The acetylation level of most lysine residues at these enzymes decreased in NF-PitNETs. (ii) Most microbe and mammalian cells depend on glycolysis to convert glucose into lactate, and produce energy in the absence of oxygen. However, most tumor cells uptake more glucose, and produce more lactate even in the presence of oxygen even though mitochondria function well, which is noted as aerobic glycolysis, or Warburg effect (30). Gluconeogenesis converts lactate or amino acids to glucose, which is the reverse pathway of glycolysis in essence (31) (**Supplementary Figure S1.2**). This study found that acetylation occurred at glycolysis/gluconeogenesis-related enzymes, including acetylation at residues K30 (only acetylated in NF-PitNETs) in 4-trimethylaminobutyraldehyde dehydrogenase (ID: P49189), K89, K5, K228, K71, and K262 (only acetylated in controls) in alpha-enolase (ID: P06733), K77 (only acetylated in NF-PitNETs) in mitochondrial pyruvate dehydrogenase E1 component subunit alpha (somatic form) (ID: P08559), K135, and K89 (only acetylated in controls) in pyruvate kinase PKM (ID: P14618), K194 (ratio of T/N =1.74, *p* = 4.24E-03), and K117 (ratio of T/N=0.25, *p*=2.50E-02) in glyceraldehyde-3-phosphate dehydrogenase (ID: P04406), K7 (only acetylated in controls) in alcohol dehydrogenase class-3 (ID: P11766), K39 (ratio of T/N=0.06, *p*=7.09E-04) in phosphoglycerate mutase 1 (ID: P18669), K291, K146, K156, and K361 (only acetylated in controls) in phosphoglycerate kinase 1 (ID: P00558), K231 (only acetylated in controls), and K225 (ratio of T/N=0.44, *p*=3.28E-04) in triosephosphate isomerase (ID: P60174) and K330 and K200 (only acetylated in controls) in fructose-bisphosphate aldolase A (ID: P04075), and K267 (ratio of T/N =1.95, *p* =1.42E-04) in mitochondrial dihydrolipoylde hydrogenase (ID: P09622). The acetylation levels at more than 4/5 lysine residues in these enzymes enriched in glycolysis/gluconeogenesis pathways were decreased in NF-PitNETs, which might result in the convert of glycolysis to the aerobic glycolysis in NF-PitNETs, and further affect tumor progression. (iii) Pyruvate, the end product of glycolysis, is reduced to lactate in cytoplasm or transport into mitochondria to enter TCA cycle for full oxidation for ATP production, and sit at the switch point between these two important carbohydrate metabolism pathways (**Supplementary Figure S1.3**) (32, 33). Pyruvate kinase (ID: P14618) is the rate-limiting enzyme at the last step of glycolysis to catalyze phosphoenolpyruvate to pyruvate. Its acetylation levels at residues K135 and K89 were decreased in NF-PitNETs (only acetylated in controls) in this study. Mitochondrial dihydrolipoyl dehydrogenase (ID: P09622) and mitochondrial pyruvate dehydrogenase E1 component subunit alpha (somatic form) (ID: P08559) are two components to form pyruvate dehydrogenase complex, which is able to catalyze pyruvate to acetyl-CoA for entering TCA cycle. Their acetylation levels at corresponding residues K267 (ratio of T/N=1.95, *p*=1.42E-04) and K77 (only acetylated in NF-PitNETs) were increased in NF-PitNETs in this study. In addition, this study found acetylation occurred at other enzymes enriched on pyruvate metabolism pathway, including acetylation at residues K30 (only acetylated in NF-PitNETs) in 4-trimethylaminobutyraldehyde dehydrogenase (ID: P49189), K314 (ratio of T/N =2.71, *p*=2.20E-02), K185 (only acetylated in NF-PitNETs), K324 and K239 (only acetylated in controls) in mitochondrial malate dehydrogenase (ID: P40926), K298, and K236 (only acetylated in controls) in cytoplasmic malate dehydrogenase (ID: P40925), and K202 (ratio of T/N=4.13, *p*=3.34E-02), K257 (ratio of T/N=0.35, *p*=1.13E-02) and K124 (only acetylated in controls) in mitochondrial acetyl-CoA acetyltransferase (ID: P24752). (iv) TCA cycle is the final metabolic pathway of carbohydrates, lipids, and amino acids, which is the central route for cellular oxidative phosphorylation and provides precursors for many anabolic pathways (34) (**Supplementary Figure S1.4**). TCA cycle occurs in mitochondria. This study found that acetylation occurred at the four enzymes residing in mitochondria, including acetylation at residues K77 (only acetylated in NF-PitNETs) in mitochondrial pyruvate dehydrogenase E1 component subunit alpha (somatic form) (ID: P08559), K314 (ratio of T/N =2.71, *p*=2.20E-02), K185 (only acetylated in NF-PitNETs), K324 and K239 (only acetylated in controls) in mitochondrial malate dehydrogenase (ID: P40926), K267 (ratio of T/N=1.95, *p*=1.42E-04) in mitochondrial dihydrolipoylde hydrogenase (ID: P09622), and K538 (only acetylated in NF-PitNETs) in mitochondrial succinate dehydrogenase [ubiquinone] flavoprotein subunit (ID: P31040). The acetylation levels at most lysine residues in these enzymes were increased in NF-PitNETs. In addition, this study found acetylation occurred at other cytoplasmic enzymes enriched on TCA cycle, including acetylation at residues at K81 (ratio of T/N =5.38, *p*=3.75E-03) in cytoplasmic isocitrate dehydrogenase [NADP] (ID: O75874), and K298, and K236 (only acetylated in controls) in cytoplasmic malate dehydrogenase (ID: P40925). (v) Glyoxylate is a highly toxic substance because it is able to be rapidly oxidized to oxalate that forms insoluble crystals with calcium, which precipitates in various organs, especially the kidneys to cause renal failure. Therefore, glyoxylate metabolism in human mainly referred to glyoxylate detoxification (35). Dicarboxylate, also called dicarboxylic acids, included oxaloacetic acid, malic acid, and aspartic acid, which participated in many metabolism pathway such as ω-oxidation of fatty acids, TCA cycle, etc. (36) (**Supplementary Figure S1.5**). This study found that acetylation occurred at glyoxylate and dicarboxylate metabolism-related molecules, including acetylation at residues K314 (ratio of T/N = 2.71, *p*=2.20E-02), K185 (only acetylated in NF-PitNETs), K324 and K239 (only acetylated in controls) in mitochondrial malate dehydrogenase (ID: P40926), K298, and K236 (only acetylated in controls) in cytoplasmic malate dehydrogenase (ID: P40925), K169 (only acetylated in controls) in catalase (ID: P04040), K202 (ratio of T/N =4.13, *p*=3.34E-02), K257 (ratio of T/N=0.35, *p*=1.13E-02), and K124 (only acetylated in controls) in mitochondrial acetyl-CoA acetyltransferase (ID: P24752), and K267 (ratio of T/N =1.95, *p*=1.42E-04) in mitochondrial dihydrolipoylde hydrogenase (ID: P09622). (vi) Valine, leucine and isoleucine, also known as branched-chain amino acids, are essential amino acids that have to be derived from diet, which can be oxidized in skeletal muscle for energy supply when exercise, and are also preferentially used by many tumor cells for protein synthesis and energy purposes. It is reported that many enzymes that catalyzed the degradation of valine, leucine and isoleucine were overexpressed in many cancers (37–40) (**Supplementary Figure S1.6**). This study found acetylation occurred at valine, leucine and isoleucine degradation-related molecules, including acetylation at residues K48 (ratio of T/N=2.97, *p*= 6.90E-03) in mitochondrial hydroxymethylglutaryl-CoA lyase (ID: P35914), K294 (only acetylated in NF-PitNETs) in mitochondrial 2-oxoisovalerate dehydrogenase subunit beta (ID: P21953), K30 (only acetylated in NF-PitNETs) in 4-trimethylaminobutyraldehyde dehydrogenase (ID: P49189), K204 (only acetylated in NF-PitNETs) in mitochondrial methylglutaconyl-CoA hydratase (ID: Q13825), K202 (ratio of T/N=4.13, *p*=3.34E-02), K257 (ratio of T/N=0.35, *p*=1.13E-02), and K124 (only acetylated in controls) in mitochondrial acetyl-CoA acetyltransferase (ID: P24752), and K267 (ratio of T/N=1.95, *p*=1.42E-04) in mitochondrial dihydrolipoylde hydrogenase (ID: P09622). The acetylation levels of most lysine residues at enzymes enriched in the valine, leucine and isoleucine degradation pathway increased in NF-PitNETs. (vii) Oxidative phosphorylation is the primary pathway for ATP synthesis and responsible for setting and maintaining metabolic homeostasis (41). It is reported that oxidative phosphorylation levels were abnormally altered in many cancers (42, 43) (**Supplementary Figure S1.7**). Except the decreased acetylation levels of residues K86 (ratio of T/N=0.36, *p*=7.61E-04) in mitochondrial cytochrome c oxidase subunit 5B (ID: P10606) and K21 (ratio of T/N=0.38, *P*=2.11E-03) in mitochondrial ATP synthase subunit epsilon (ID: P56381), the acetylation levels of all other lysine residues in enzymes enriched in oxidative phosphorylation pathway were increased in NF-PitNETs, including residues K538 (only acetylated in NF-PitNETs) in mitochondrial succinate dehydrogenase [ubiquinone] flavoprotein subunit (ID: P31040), K233, K154, and K244 (only acetylated in NF-PitNETs) in mitochondrial ATP synthase F(0) complex subunit B1 (ID: P24539), K522 (only acetylated in NF-PitNETs) in mitochondrial ATP synthase subunit beta (ID: P06576), K60 (ratio of T/N=3.79, *p*=4.00E-02), K199 and K162 (only acetylated in NF-PitNETs) in mitochondrial ATP synthase subunit O (ID: P48047), K136 (only acetylated in NF-PitNETs) in mitochondrial ATP synthase subunit delta (ID: P30049), K539 (ratio of T/N=1.77, *p*=4.68E-02), and K427 (only acetylated in NF-PitNETs) in mitochondrial ATP synthase subunit alpha (ID: P25705), K63 (ratio of T/N=5.48, *p*=9.47E-05), and K72 (ratio of T/N= 2.94, *p*=2.02E-03) in mitochondrial ATP synthase subunit d (ID: O75947), and K37 (ratio of T/N=1.61, *p*=1.19E-02) in mitochondrial ATP synthase subunit epsilon (ID: P56381). (viii) Metabolic pathway involves many interconnected cellular pathways that ultimately provide cells with energy required to execute their function (44). In cancer, oncogene activation and tumor suppressor loss promote metabolic reprogramming, and cause enhanced nutrient uptake to supply malignant cell energetic and biosynthetic pathways (45). Therefore, metabolic pathway alteration, in another word, metabolic reprogramming, might also be key for NF-PitNETs tumorigenesis and progression (**Supplementary Figure S1.8**). This study found that metabolic pathway enriched the largest number of DAPs (n = 34), and acetylation occurred at the metabolic pathway-related molecules, including acetylation at residues K89, K5, K228, K71, and K262 (only acetylated in controls) in alpha-enolase (ID: P06733), K77 (only acetylated in NF-PitNETs) in mitochondrial pyruvate dehydrogenase E1 component subunit alpha (somatic form) (ID: P08559), K135, and K89 (only acetylated in controls) in pyruvate kinase PKM (ID: P14618), K7 (ratio of T/N=0.58, *p*=1.52E-02) in transaldolase (ID: P37837), K194 (ratio of T/N=1.74, *p*=4.24E-03), and K117 (ratio of T/N=0.25, *p*=2.50E-02) in glyceraldehyde-3-phosphate dehydrogenase (ID: P04406), K81 (ratio of T/N=5.38, *p*=3.75E-03) in cytoplasmic isocitrate dehydrogenase [NADP] (ID: O75874), K314 (ratio of T/N =2.71, *p*=2.20E-02), K185 (only acetylated in NF-PitNETs), K324 and K239 (only acetylated in controls) in mitochondrial malate dehydrogenase (ID: P40926), K298, and K236 (only acetylated in controls) in cytoplasmic malate dehydrogenase (ID: P40925), K7 (only acetylated in controls) in alcohol dehydrogenase class-3 (ID: P11766), K39 (ratio of T/N=0.06, *p*=7.09E-04) in phosphoglycerate mutase 1 (ID: P18669), K302 (ratio of T/N=1.71, *p*=8.69E-03) in mitochondrial aspartate aminotransferase (ID: P00505), K291, K146, K156 and K361 (only acetylated in controls) in phosphoglycerate kinase 1 (ID: P00558), K538 (only acetylated in NF-PitNETs) in mitochondrial succinate dehydrogenase [ubiquinone] flavoprotein subunit (ID: P31040), K231(only acetylated in controls), and K225 (ratio of T/N=0.44, *p*=3.28E-04) in triosephosphate isomerase (ID: P60174), and K330 and K200 (only acetylated in controls) in fructose-bisphosphate aldolase A (ID: P04075), K202 (ratio of T/N =4.13, *p*=3.34E-02), K257 (ratio of T/N=0.35, *p*=1.13E-02), and K124 (only acetylated in controls) in mitochondrial acetyl-CoA acetyltransferase (ID: P24752), K267 (ratio of T/N=1.95, *p*=1.42E-04) in mitochondrial dihydrolipoylde hydrogenase (ID: P09622), K63 (ratio of T/N= 5.48, *p*=9.47E-05), and K72 (ratio of T/N= 2.94, *p*=2.02E-03) in mitochondrial ATP synthase subunit d (ID: O75947), K30 (only acetylated in NF-PitNETs) in 4-trimethylaminobutyraldehyde dehydrogenase (ID: P49189), K539 (ratio of T/N=1.77, *p*=4.68E-02 ), and K427 (only acetylated in NF-PitNETs) in mitochondrial ATP synthase subunit alpha (ID: P25705), K233, K154, and K244 (only acetylated in NF-PitNETs) in mitochondrial ATP synthase F(0) complex subunit B1 (ID: P24539), K522 (only acetylated in NF-PitNETs) in mitochondrial ATP synthase subunit beta (ID: P06576), K60 (ratio of T/N = 3.79, *p*= 4.00E-02), K199 and K162 (only acetylated in NF-PitNETs) in mitochondrial ATP synthase subunit O (ID: P48047), K86 (ratio of T/N=0.36, *p*=7.61E-04) in mitochondrial cytochrome c oxidase subunit 5B (ID: P10606), K37 (ratio of T/N=1.61, *p*=1.19E-02), and K21 (ratio of T/N=0.38, *p*=2.11E-03) in mitochondrial ATP synthase subunit epsilon (ID: P56381), K294 (only acetylated in NF-PitNETs) in mitochondrial 2-oxoisovalerate dehydrogenase subunit beta (ID: P21953), K26 (only acetylated in controls) in nucleoside diphosphate kinase A (ID: P15531), K70 (ratio of T/N=13.46, *p* = 1.97E-03) in mitochondrial NAD(P) transhydrogenase (ID: Q13423), K1704 (ratio of T/N = 2.28, *p* = 6.37E-03) in fatty acid synthase (ID: P49327), K189 (only acetylated in NF-PitNETs) in mitochondrial monofunctional C1-tetrahydrofolate synthase (ID: Q6UB35), K419 (ratio of T/N=13.46, *p*=5.17E-03) in retinal dehydrogenase 1 (ID: P00352), K48 (ratio of T/N=2.97, *p*=6.90E-03) in mitochondrial hydroxymethylglutaryl-CoA lyase (ID: P35914), K204 (only acetylated in NF-PitNETs) in mitochondrial methylglutaconyl-CoA hydratase (ID: Q13825), and K136 (only acetylated in NF-PitNETs) in mitochondrial ATP synthase subunit delta (ID: P30049). These acetylated proteins and acetylation sites involved in the metabolic pathway would expand the research data for the study of NF-PitNETs pathogenesis. (ix) Biosynthesis of amino acids is a crucial process constructing the precursor of proteins to participate in vital movement. The deregulated catabolism/anabolism of amino acids, especially serine, glutamine and glycine, were reported to function as metabolic regulators in promoting cancer cell growth (46) (**Supplementary Figure S1.9**). The acetylated proteins enriched in this pathway mainly consisted of enzymes involved in glycolysis, which were able to catalyze syntheses of 3-hosphoglycerate, phosphoenolpyruvate, and pyruvic acid, and provided carbon skeleton for serine, tyrosine and alanine, etc. This study found acetylation occurred at the enzymes involved in biosynthesis of amino acids, including acetylation at residues K89, K5, K228, K71, and K262 (only acetylated in controls) in alpha-enolase (ID: P06733), K135, and K89 (only acetylated in controls) in pyruvate kinase PKM (ID: P14618), K7 (ratio of T/N=0.58, *p*=1.52E-02) in transaldolase (ID: P37837), K194 (ratio of T/N=1.74, *p*=4.24E-03), and K117 (ratio of T/N=0.25, *p*=2.50E-02) in glyceraldehyde-3-phosphate dehydrogenase (ID: P04406), K81 (ratio of T/N=5.38, *p*=3.75E-03) in cytoplasmic isocitrate dehydrogenase [NADP] (ID: O75874), K39 (ratio of T/N =0.06, *p*=7.09E-04) in phosphoglycerate mutase 1 (ID: P18669), K302 (ratio of T/N=1.71, *p*=8.69E-03) in mitochondrial aspartate aminotransferase (ID: P00505), K291, K146, K156 and K361 (only acetylated in controls) in phosphoglycerate kinase 1 (ID: P00558), K231 (only acetylated in controls), and K225 (ratio of T/N=0.44, *p*=3.28E-04) in triosephosphate isomerase (ID: P60174), and K330 and K200 (only acetylated in controls) in fructose-bisphosphate aldolase A (ID: P04075).

Three pathways associated with nervous system diseases were Parkinson’s disease pathway, Huntington’s disease pathway, and Alzheimer’s disease pathway. The acetylated proteins enriched in these three pathways were mainly enzymes involved in metabolism and energy yield, which indicated that the acetylation mainly occurred at enzymes, and their alterations might result in extensive influence in metabolism by various pathway. (i) This study found acetylation occurred at the Parkinson’s disease pathway-related molecules (**Supplementary Figure S1.10**), including acetylation at residues K538 (only acetylated in NF-PitNETs) in mitochondrial succinate dehydrogenase [ubiquinone] flavoprotein subunit (ID: P31040), K63 (ratio of T/N=5.48, *p*=9.47E-05), and K72 (ratio of T/N=2.94, *p*=2.02E-03) in mitochondrial ATP synthase subunit d (ID: O75947), K539 (ratio of T/N=1.77, *p*=4.68E-02), and K427 (only acetylated in NF-PitNETs) in mitochondrial ATP synthase subunit alpha (ID: P25705), K233, K154, and K244 (only acetylated in NF-PitNETs) in mitochondrial ATP synthase F(0) complex subunit B1 (ID: P24539), K522 (only acetylated in NF-PitNETs) in mitochondrial ATP synthase subunit beta (ID: P06576), K60 (ratio of T/N=3.79, *p*=4.00E-02), K199 and K162 (only acetylated in NF-PitNETs) in mitochondrial ATP synthase subunit O (ID: P48047), K86 (ratio of T/N=0.36, *p*=7.61E-04) in mitochondrial cytochrome c oxidase subunit 5B (ID: P10606), K37 (ratio of T/N=1.61, *p*= 1.19E-02), and K21 (ratio of T/N=0.38, *p*=2.11E-03) in mitochondrial ATP synthase subunit epsilon (ID:P56381), and K136 (only acetylated in NF-PitNETs) in mitochondrial ATP synthase subunit delta (ID: P30049), K195 (only acetylated in controls) in ubiquitin carboxyl-terminal hydrolase isozyme L1 (ID: P09936), K72 (only acetylated in controls), and K427 (only acetylated in NF-PitNETs) in voltage-dependent anion-selective channel protein 2 (ID: P45880), K23 (only acetylated in NF-PitNETs) in ADP/ATP translocase 3 (ID: P12236), K23 (only acetylated in NF-PitNETs) in ADP/ATP translocase 1 (ID: P12235), K62 (only acetylated in controls) in protein deglycase DJ-1 (ID: Q99497), and K23 (only acetylated in NF-PitNETs) ADP/ATP translocase 2 (ID: P05141). (ii) This study found acetylation occurred at the Huntington’s disease pathway-related molecules (**Supplementary Figure S1.11**), including acetylation at residues K538 (only acetylated in NF-PitNETs) in mitochondrial succinate dehydrogenase [ubiquinone] flavoprotein subunit (ID: P31040), K63 (ratio of T/N=5.48, *p*=9.47E-05), and K72 (ratio of T/N= 2.94, *p*=2.02E-03) in mitochondrial ATP synthase subunit d (ID: O75947), K539 (ratio of T/N=1.77, *p*=4.68E-02 ), and K427 (only acetylated in NF-PitNETs) in mitochondrial ATP synthase subunit alpha (ID: P25705), K233, K154, and K244 (only acetylated in NF-PitNETs) in mitochondrial ATP synthase F(0) complex subunit B1 (ID: P24539), K522 (only acetylated in NF-PitNETs) in mitochondrial ATP synthase subunit beta (ID: P06576), K60 (ratio of T/N=3.79, *p*=4.00E-02), K199 and K162 (only acetylated in NF-PitNETs) in mitochondrial ATP synthase subunit O (ID: P48047), K86 (ratio of T/N=0.36, *p*=7.61E-04) in mitochondrial cytochrome c oxidase subunit 5B (ID: P10606), K37 (ratio of T/N=1.61, *p*=1.19E-02), and K21 (ratio of T/N=0.38, *p*=2.11E-03) in mitochondrial ATP synthase subunit epsilon (ID: P56381), and K136 (only acetylated in NF-PitNETs) in mitochondrial ATP synthase subunit delta (ID: P30049), K72 (only acetylated in controls), and K427 (only acetylated in NF-PitNETs) in voltage-dependent anion-selective channel protein 2 (ID: P45880), K23 (only acetylated in NF-PitNETs) in ADP/ATP translocase 3 (ID: P12236), K23 (only acetylated in NF-PitNETs) in ADP/ATP translocase 1 (ID: P12235), K23 (only acetylated in NF-PitNETs) ADP/ATP translocase 2 (ID: P05141), K123 (ratio of T/N=0.30, *p*=1.38E-03) in superoxide dismutase [Cu-Zn] (ID: P00441), and K130 (only acetylated in NF-PitNETs) in mitochondrial superoxide dismutase [Mn] (ID: P04179). (iii) This study found acetylation occurred at the Alzheimer’s disease pathway-related molecules (**Supplementary Figure S1.12**), including acetylation at residues K538 (only acetylated in NF-PitNETs) in mitochondrial succinate dehydrogenase [ubiquinone] flavoprotein subunit (ID: P31040), K63 (ratio of T/N=5.48, *p*=9.47E-05), and K72 (ratio of T/N= 2.94, *p*=2.02E-03) in mitochondrial ATP synthase subunit d (ID: O75947), K539 (ratio of T/N=1.77, *p*=4.68E-02 ), and K427 (only acetylated in NF-PitNETs) in mitochondrial ATP synthase subunit alpha (ID: P25705), K233, K154, and K244 (only acetylated in NF-PitNETs) in mitochondrial ATP synthase F(0) complex subunit B1 (ID: P24539), K522 (only acetylated in NF-PitNETs) in mitochondrial ATP synthase subunit beta (ID: P06576), K60 (ratio of T/N=3.79, *p*=4.00E-02), K199 and K162 (only acetylated in NF-PitNETs) in mitochondrial ATP synthase subunit O (ID: P48047), K86 (ratio of T/N=0.36, *p*=7.61E-04) in mitochondrial cytochrome c oxidase subunit 5B (ID: P10606), K37 (ratio of T/N=1.61, *p*=1.19E-02), and K21 (ratio of T/N=0.38, *p*=2.11E-03) in mitochondrial ATP synthase subunit epsilon (ID: P56381), and K136 (only acetylated in NF-PitNETs) in mitochondrial ATP synthase subunit delta (ID: P30049), K194 (ratio of T/N=1.74, *p*=4.24E-03), and K117 (ratio of T/N=0.25, *p*=2.50E-02) in glyceraldehyde-3-phosphate dehydrogenase (ID: P04406), and K133 (only acetylated in controls) in calmodulin (Fragment) (ID: H0Y7A7).

Three pathways pathogenic *Escherichia Coli* infection pathway, viral carcinogenesis, and African trypanosomiasis were associated with infectious diseases. (i) The acetylated proteins enriched in pathogenic *Escherichia Coli* infection pathway extensively existed in cytoplasm and nucleus. Tubulin, actin, and ezrin constituted cytoskeleton, maintained cell morphology and motility, and regulated cell cycle or cell-cell adhesion. Nucleolin participated in the cleavage of rRNA precursors, DNA replication, and cell cycle regulation (47) (**Supplementary Figure S1.13**). This study found that acetylation occurred at the pathogenic *Escherichia Coli* infection-related molecules, including acetylation at residues K449 (only acetylated in controls) in nucleolin (ID: P19338), K440 (only acetylated in controls) in tubulin alpha-1C chain (ID: F5H5D3), K61 (ratio of T/N=0.22, *p*=2.59E-04) and K326 (only acetylated in controls) in actin cytoplasmic 1 (ID: P60709), K394 (ratio of T/N=0.29, *p* =2.08E-02) and K336 (ratio of T/N=0.21, *p*=1.77E-04) in tubulin alpha-1B chain (ID: P68363), and K35 (only acetylated in controls) in ezrin (ID: P15311). (ii) One of proteins enriched in viral carcinogenesis pathway is 14-3-3 protein. 14-3-3 protein has many subtypes, including 14-3-3 protein gamma, theta, and epsilon, and many of them have carcinogenic potential (48–50) (**Supplementary Figure S1.14**). This study found that acetylation occurred at the viral carcinogenesis-related molecules, including acetylation at residues K135 and K89 (only acetylated in controls) in pyruvate kinase (ID: P14618), K152 (only acetylated in controls) in 14-3-3 protein gamma (ID: P61981), K9 (ratio of T/N=1.60, *p*=2.36E-02) in histone H4 (ID: P62805), K398 (ratio of T/N=0.40, *p*=4.72E-02) in alpha-actinin-1 (ID: P12814), K47 (only acetylated in controls) in histone H2B type 1-K (ID: O60814), K68 (only acetylated in controls) in 14-3-3 protein theta (ID: P27348), K150 (only acetylated in controls) in ran-specific GTPase-activating protein (ID: P43487), and K3 and K9 (only acetylated in controls) in 14-3-3 protein zeta/delta (ID: P63104). The acetylation level decreased in 14-3-3 protein in NF-PitNETs, the alteration of which might support NF-PitNETs tumorigenesis. (iii) Human African trypanosomiasis is a potentially fatal disease caused by the *Trypanosoma Brucei* sp (a kind of parasite) (51) (**Supplementary Figure S1.15**). This study found that acetylation occurred at the African trypanosomiasis-related molecules, including acetylation at residues K12 (ratio of T/N=0.37, *p*=4.29E-02) and K91 (ratio of T/N=0.13, *p*=1.13E-02) in hemoglobin subunit alpha (ID: P69905), K96 (only acetylated in controls) in mutant hemoglobin beta chain (Fragment) (ID: Q9BWU5), K57 (only acetylated in controls) in hemoglobin alpha-1 globin chain (Fragment) ID: E9M4D4), K12 (ratio of T/N=0.37, *p* =4.29E-02) in alpha globin chain (Fragment) (ID: U3PXP0), K96 (ratio of T/N=0.54, *p*=2.14E-02) in hemoglobin subunit beta (ID: P68871), K157 (ratio of T/N=0.18, *p*=3.58E-03), K262, K230, and K206 (only acetylated in controls) in apolipoprotein A-I (ID: P02647), and K17 (ratio of T/N=0.62, *p*=2.01E-02) in alpha-2 globin chain (ID: D1MGQ2).

In recent years, mammalian immune cells were found to synthesize antibiotics, itaconic acid, from citric acid cycle intermediate, to prevent bacteria from surviving in cells (52) (**Supplementary Figure S1.16**). All proteins enriched in biosynthesis of antibiotic pathway were also enriched in the nine metabolism and energy yield pathways. This study found that acetylation occurred at the biosynthesis of antibiotics-related molecules, including acetylation at residues K89, K5, K228, K71, and K262 (only acetylated in controls) in alpha-enolase (ID: P06733), K77 (only acetylated in NF-PitNETs) in mitochondrial pyruvate dehydrogenase E1 component subunit alpha (somatic form) (ID: P08559), K26 (only acetylated in controls) in nucleoside diphosphate kinase A (ID: P15531), K135, and K89 (only acetylated in controls) in pyruvate kinase PKM (ID: P14618), K7 (ratio of T/N=0.58, *p*=1.52E-02) in transaldolase (ID: P37837), K194 (ratio of T/N=1.74, *p*=4.24E-03), and K117 (ratio of T/N=0.25, *p*=2.50E-02) in glyceraldehyde-3-phosphate dehydrogenase (ID: P04406), K294 (only acetylated in NF-PitNETs) in mitochondrial 2-oxoisovalerate dehydrogenase subunit beta (ID: P21953), K81 (ratio of T/N=5.38, *p*=3.75E-03) in isocitrate dehydrogenase [NADP] cytoplasmic (ID: O75874), K314 (ratio of T/N=2.71, *p*=2.20E-02), K185 (only acetylated in NF-PitNETs), K324 and K239 (only acetylated in controls) in mitochondrial malate dehydrogenase (ID: P40926), K298, and K236 (only acetylated in controls) in cytoplasmic malate dehydrogenase (ID: P40925), K7 (only acetylated in controls) in alcohol dehydrogenase class-3 (ID: P11766), K39 (ratio of T/N=0.06, *p*=7.09E-04) in phosphoglycerate mutase 1 (ID: P18669), K302 (ratio of T/N=1.71, *p*=8.69E-03) in mitochondrial aspartate aminotransferase (ID: P00505), K291, K146, K156 and K361 (only acetylated in controls) in phosphoglycerate kinase 1 (ID: P00558), K538 (only acetylated in NF-PitNETs) in mitochondrial succinate dehydrogenase [ubiquinone] flavoprotein subunit (ID: P31040), K231 (only acetylated in controls), and K225 (ratio of T/N=0.44, *p*=3.28E-04) in triosephosphate isomerase (ID: P60174), K330 and K200 (only acetylated in controls) in fructose-bisphosphate aldolase A (ID: P04075), K202 (ratio of T/N=4.13, *p*=3.34E-02), K257 (ratio of T/N=0.35, *p*=1.13E-02), and K124 (only acetylated in controls) in mitochondrial acetyl-CoA acetyltransferase (ID: P24752), and K267 (ratio of T/N=1.95, *p*=1.42E-04) in mitochondrial dihydrolipoylde hydrogenase (ID: P09622), K30 (only acetylated in NF-PitNETs) in 4-trimethylaminobutyraldehyde dehydrogenase (ID: P49189), and K169 (only acetylated in controls) in catalase (ID: P04040). The acetylation levels of most of these proteins were decreased in NF-PitNETs.

Peroxisome is consisted of many kinds of oxidases, and contributes to cellular lipid metabolism and redox balance. Peroxisome has ability of detoxification, including removal of superoxide radicals originated from respiratory chain (53). The dysfunction of peroxisome is associated with the development of many cancers (54–56) (**Supplementary Figure S1.17**). This study found that acetylation occurred at the peroxisome complex, including acetylation at residues K48 (ratio of T/N=2.97, *p*=6.90E-03) in mitochondrial hydroxymethylglutaryl-CoA lyase (ID: P35914), K81 (ratio of T/N=5.38, *p*=3.75E-03) in isocitrate dehydrogenase [NADP] cytoplasmic (ID: O75874), K130 (ratio of T/N=0.31, *p*=3.31E-03), and K169 (only acetylated in controls) in peroxiredoxin-1 (ID: Q06830), K169 (only acetylated in controls) in catalase (ID: P04040), K123 (ratio of T/N=0.30, *p*=1.38E-03) in superoxide dismutase [Cu-Zn] (ID: P00441), and K130 (only acetylated in NF-PitNETs) in mitochondrial superoxide dismutase [Mn] (ID: P04179).

The last pathway was complement and coagulation cascade pathway, and all these DAPs enriched in this pathway were from blood. In the blood circulation, the coagulation system, platelets, complement system, and fibrinolysis system constructed a close and complex network. They activated and regulated each other, and mutually mediated tissue homeostasis and immune monitoring. The deregulation of each cascade system caused clinical manifestations and the progression of different diseases, such as C3 glomerulonephritis, sepsis, and systemic lupus erythematosus (57) (**Supplementary Figure S1.18**). This study found that acetylation occurred at the complement and coagulation cascade-related molecules, including acetylation at residues K148, K620, K167, and K195 (only acetylated in NF-PitNETs) in fibrinogen alpha chain (ID: P02671), K153 (only acetylated in NF-PitNETs) and K231 (only acetylated in controls) in fibrinogen gamma chain (ID: P02679), K77 (only acetylated in NF-PitNETs) in fibrinogen beta chain (ID: P02675), K298 (ratio of T/N=0.07, *p*=2.05E-03) and K153 (only acetylated in controls) in alpha-1-antitrypsin (ID: P01009), and K1176 (only acetylated in controls) in alpha-2-macroglobulin (ID: P01023).

### Integration of Acetylomics and Ubiquitinomics Data in NF-PitNETs *Versus* Controls

A total of 15 lysine sites within 14 proteins was modified by both acetyl group and ubiquitin (**Table 1**). Of them, histone H2A type 1 (P04908), histone H2A (A0A0U1RRH7), and histone H2B (B4DR52) were histone to constitute nucleosome, whose main molecular functions were DNA binding and protein heterodimerization. histone H2A type 1 (P04908) and histone H2A (A0A0U1RRH7) maintained the structure of chromatin and their silence repressed transcription (58). Histone H2A type 1 (P04908) negatively regulated cell proliferation. Epididymis luminal protein 112 (B2RDW1) had two lysine sites that were both acetylated and ubiquitinated, which contributed to form the complex structure of ribosome and participated in translation - a cellular metabolic process to form proteins. Vimentin (P08670) attached to the nucleus, mitochondria, and endoplasmic reticulum was found in various cells, especially mesenchymal cells. Vimentin (P08670) had extensive molecular functions; for example, vimintin (P08670) bound scaffold proteins to activate and localize signaling components to specific areas of cell (59, 60), also participated in SMAD protein signal transduction that was the key step of TGF-β pathway regulating cell proliferation, differentiation, migration, and death (61). Ubiquitin carboxyl-terminal hydrolase (D6R956) facilitated protein deubiquitination to affect protein catabolism (62). Vesicle-associated membrane protein 2 (L7N2F9) mediated membrane fusion, which was a basic step of many biological processes, such as neurotransmitter transmission and antigen presenting. Growth hormone A1 (Q5I0G2) was coded by PRL gene, and regulated the hormone activity. Actin cytoplasmic 1 (P60709) localized in the cytoplasm and nucleus, and participated in cytoskeleton formation, cell motility, gene transcription, and repair of damaged DNA (63, 64). Tubulin alpha-1C chain 1 (F5H5D3) was a member of tubulin superfamily, and played functions in cytoskeleton maintaining and spindle fiber constitution in mitosis. Alpha-2 globin chain (D1MGQ2), hemoglobin subunit beta (P68871), hemoglobin subunit alpha (P69905), and hemoglobin subunit delta (P02042) were the parts of hemoglobin, and played roles in oxygen transport, hemoglobin formation, and cellular oxidant detoxification. Thereby, these proteins that were both acetylated and ubiquitinated at the same site in NF-PitNETs were involved in multiple biological processes, including gene expression, protein metabolic process, cell motility, oxygen transport, and hemostatic process. Furthermore, comparative analysis of these proteins (D1MGQ2, P6887, P04908, P60709, L7N2F9, F5H5D3, B2RDW1, and Q5I0G2), which were quantified with statistically significant ratio of T/N in both acetylomics and ubiquitinomics data, found that their acetylation levels were decreased but their ubiquitination levels were increased in NF-PitNETs, which showed the competitive characteristics of acetylation and ubiquitination at the lysine site in a protein in NF-PitNETs.

**Table 1:** The proteins that were simultaneously acetylated and ubiquitinated at the same sites in NF-PitNETs and controls.

Acetylated peptides										Ubiquitinated peptides									
Accession No.	Gene name	Protein name	Modified peptide	Modified positions	Modified probabilities	Average (N)	Average (T)	Ratio (T/N)	P-value (t-test)	Accession No.	Gene name	Protein name	Modified peptides	Modified positions	Modified probabilities	Modified level (N)	Modified level (T)	Ratio (T/N)	t-test p-value
D1MGQ2	HBA2	Alpha-2 globin chain	AAWGK*VGAHAGEYGAEALER	17	1	418780000	260586667	0.62	2.01E-02	D1MGQ2	HBA2	Alpha-2 globin chain	AAWGK*VGAHAGEYGAEALER	17	1		7090000		
P68871	HBB	Hemoglobin subunit beta	GTFATLSELHCDK*LHVDPENFR	96	1	3518300000	1890233333	0.54	2.14E-02	P68871	HBB	Hemoglobin subunit beta	GTFATLSELHCDK*LHVDPENFR	96	1	18900000	91300000	4.83	1.23E-02
P04908	HIST1H2AB	Histone H2A type 1-B/E	NDEELNK*LLGR	96	1	18919333	7585433	0.40	1.25E-03	P04908	HIST1H2AB	Histone H2A type 1-B/E	NDEELNK*LLGR	96	1		3450000		
P60709	ACTB	Actin, cytoplasmic 1	DSYVGDEAQSK*R	61	1	107031000	23652333	0.22	2.59E-04	P60709	ACTB	Actin, cytoplasmic 1	DSYVGDEAQSK*R	61	1		2050000		
A0A0U1RRH7		Histone H2A	NDEELNK*LLGK	96	1	17805333	3620733	0.20	3.35E-05	A0A0U1RRH7		Histone H2A	NDEELNK*LLGK	96	0.996				
P69905	HBA1	Hemoglobin subunit alpha	TYFPHFDLSHGSAQVK*GHGK	57	1	319430000	425226667	1.33	9.01E-02	P69905	HBA1	Hemoglobin subunit alpha	TYFPHFDLSHGSAQVK*	57	1	1940000	206000000	106.3	5.10E-03
D6R956	UCHL1	Ubiquitin carboxyl-terminal hydrolase	CFEK*NEAIQAAHDAVAQEGQCR	135	1	10472100	7052300	0.67	1.84E-01	D6R956	UCHL1	Ubiquitin carboxyl-terminal hydrolase	CFEK*NEAIQAAHDAVAQEGQCR	135	1		5860000		
P02042	HBD	Hemoglobin subunit delta	GTFSQLSELHCDK*LHVDPENFR	96	1	2649686667	964430000	0.36	5.16E-01	P02042	HBD	Hemoglobin subunit delta	GTFSQLSELHCDK*LHVDPENFR	96	1	15700000	20100000	1.28	1.43E-01
P08670	VIM	Vimentin	QVDQLTNDK*AR	168	1	7992500				P08670	VIM	Vimentin	QVDQLTNDK*AR	168	1	1990000	2970000	1.49	2.74E-01
L7N2F9		Uncharacterized protein	ADALQAGASQFETSAAK*LK	83	1	10451000				L7N2F9		Uncharacterized protein	ADALQAGASQFETSAAK*LK	83	0.876		20100000		
F5H5D3	TUBA1C	Tubulin alpha-1C chain1	VGINYQPPTVVPGGDLAK*VQR	370	1	21804500				F5H5D3	TUBA1C	Tubulin alpha-1C chain1	VGINYQPPTVVPGGDLAK*VQR	370	1	22700000	37800000	1.67	1.38E-03
B2RDW1	RPS30A	Epididymis luminal protein 112	TITLEVEPSDTIENVK*AK	27	1	51187500				B2RDW1	RPS30A	Epididymis luminal protein 112	TITLEVEPSDTIENVK*AK	27	0.956		19500000		
Q5I0G2	PRL	Prolactin	AVEIEEQTK*R	152	1	103715667				Q5I0G2	PRL	Growth hormone A1	AVEIEEQTK*R	153	1		5210000		
B4DR52		Histone H2B	HAVSEGTK*AVTK	117	1					B4DR52		Histone H2B	HAVSEGTK*AVTK	117	1		47000000		
B2RDW1	RPS30A	Epididymis luminal protein 112	IQDK*EGIPPDQQR	33	1					B2RDW1	RPS30A	Epididymis luminal protein 112	IQDK*EGIPPDQQR	33	1	2680000	36800000	13.74	9.55E-03

### Integration of Acetylomics Data and Invasive Transcriptomics Data in NF-PitNETs *Versus* Controls

**Figure 8:**
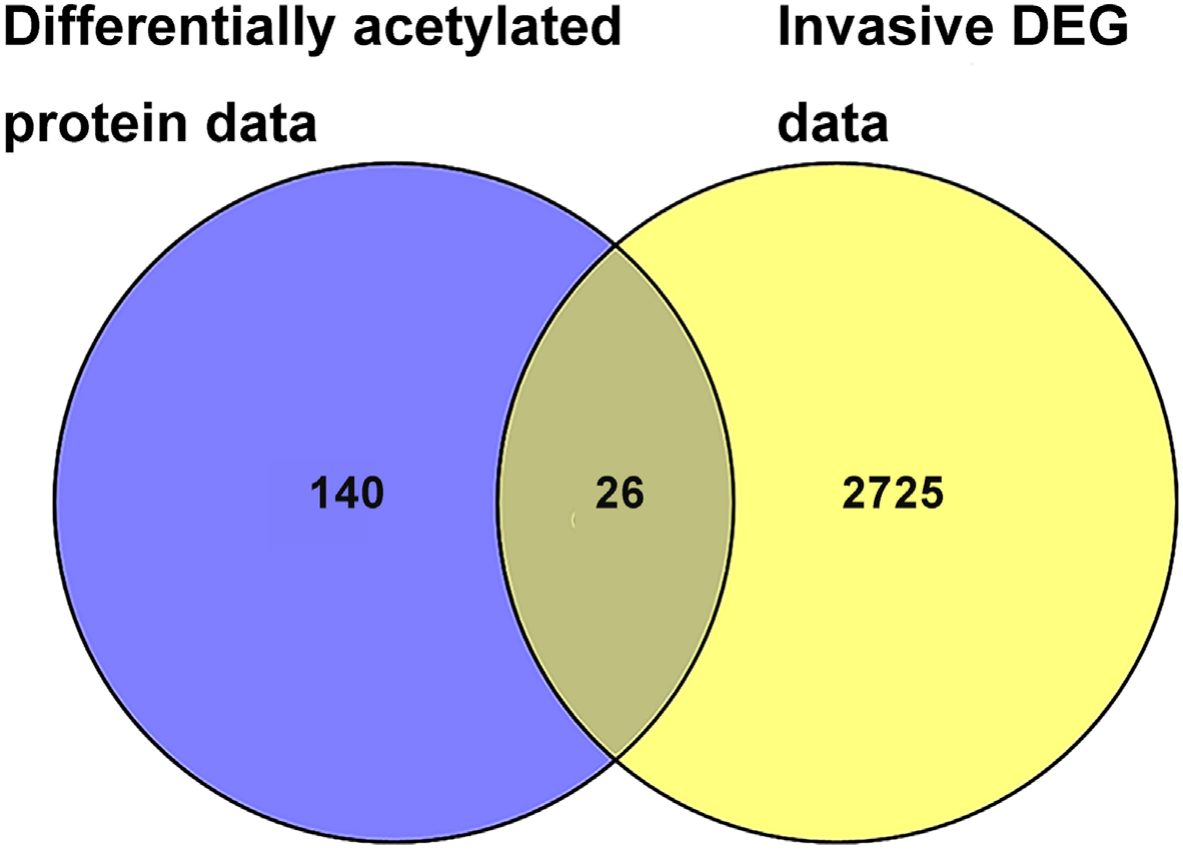
The overlapping analysis between DAP data in NF-PitNETs *vs.* controls and DEG data in invasive NF-PitNETs *vs.* controls. The invasive DEG data were mined from the GEO database.

A total of 26 overlapped molecules was identified between DAP data and invasive DEG data to investigate the effect of acetylation on the invasive behavior of NF-PitNETs (**Figure 8**; **Table 2**). These overlapped molecules (DAPs; Invasive DEGs) were enriched in eight statistically significant KEGG signaling pathways, including glycolysis/gluconeogenesis, carbon metabolism, oxidative phosphorylation, fructose and mannose metabolism, biosynthesis of amino acids, Parkinson’s disease, Alzheimer’s disease, and Huntington’s disease (**Supplementary Table 9**). GO analysis revealed that these overlapped molecules were significantly enriched in multiple MFs (**Supplementary Table 10**), CCs (**Supplementary Table 11**), and BPs (**Supplementary Table 12**). For example, TPI1 (triosephosphate isomerase) was located in extracellular space, extracellular exosome, and cytosol, performed protein binding molecular function, and participated in canonical glycolysis, glycolytic process, and gluconeogenesis. Clustering analysis of these KEGG pathways, MFs, CCs, and BPs showed that most overlapped molecules were related to metabolism and energy production (**Table 3**). Metabolic reprogramming, such as “Warburg effect”, had been recognized as a promotion mechanism for tumorigenesis and malignant activity (30, 65), and acetylation regulated the physiological functions of most metabolic enzymes (66). Thereby, the invasiveness of NF-PitNETs might be associated with acetylation-mediated metabolic reprogramming.

**Table 2:** The overlapped molecules between differentially acetylated protein (DAP) data and invasive DEG data in NF-PitNETs. LogFC = log2(Fold change).

DAP data in NF-PitNETs											DEG data in invasive NF-PitNETs						
Accession No.	Gene name	Protein name	Modified positions	Modified probabilities	Charge	*m/z*	Average (N)	Average (T)	Ratio (T/N)	P-value (t-test)	DEG name	logFC	AveExpr	*t*	p-value	Adjusted p-value	B
P60174	TPI1	Triosephosphate isomerase	225	1	2	889.5	28466667	12484667	0.44	3.28E-04	TPI1	1.7746	10.1649	6.8527	5.53E-05	2.64E-03	2.2279
			231	1	2	880.9	15229500										
Q9HCJ6	VAT1L	Synaptic vesicle membrane protein VAT-1 homolog-like	240	1	2	632.3	3193300				VAT1L	-2.8646	8.1336	-4.2145	1.97E-03	1.87E-02	-1.4591
P02787	TF	Serotransferrin	676	1	2	826.9	7204767				TF	-3.9417	8.8827	-3.5149	5.98E-03	3.63E-02	-2.5952
			546	1	2	703.9	11366000										
			37	1	3	680.0	18033333										
			60	1	2	793.4	19133000										
			659	1	3	495.6	20861333										
P11142	HSPA8	Heat shock cognate 71 kDa protein	348	1	2	746.9	2893050				HSPA8	1.3891	11.6235	4.6920	9.61E-04	1.24E-02	-0.7175
			589	1	2	894.4	9957600										
P00738	HP	Haptoglobin	321	1	2	658.8	6138650	2443767	0.40	1.66E-02	HP	-4.7051	9.9317	-5.5917	2.71E-04	6.08E-03	0.5913
P05060	CHGB	Secretogranin-1	465	1	3	512.3	12902000	3935700	0.31	2.52E-02	CHGB	-1.3288	14.5253	-3.2997	8.51E-03	4.53E-02	-2.9533
			455	1	2	630.8	7019633										
			586	1	2	898.9	7349667										
			482	1	3	927.8	7493900										
			386	1	3	667.6	7725600										
			324	1	3	787.0	15918667										
			159	1	4	631.3	28833333										
			62	1	2	437.3	39083500										
			653	1	2	709.9	41254000										
			228	1	2	902.9	57414333										
			514	1	4	347.2	171960000										
P25705	ATP5A1	ATP synthase subunit alpha, mitochondrial	539	1	2	580.8	30030333	53227000	1.77	4.68E-02	ATP5A1	1.1189	14.7620	5.8239	1.99E-04	5.20E-03	0.9097
			427	1	2	553.8		2483867									
P12235	SLC25A4	ADP/ATP translocase 1	23	1	3	695.7		5633800			SLC25A4	1.1105	13.4174	3.9198	3.12E-03	2.44E-02	-1.9315
Q6UB35	MTHFD1L	Monofunctional C1-tetrahydrofolate synthase, mitochondrial	189	1	2	828.5		1644450			MTHFD1L	-1.9847	6.1122	-4.1324	2.24E-03	2.01E-02	-1.5898
O60814	HIST1H2BK	Histone H2B type 1-K	47	1	2	775.9	14843000				HIST1H2BK	1.2059	11.2191	3.8511	3.48E-03	2.60E-02	-2.0430
P01189	POMC	Pro-opiomelanocortin	215	1	3	992.5	14740667				POMC	-7.4874	13.4887	-3.8110	3.71E-03	2.70E-02	-2.1083
P02768	ALB	Serum albumin	198	1	2	1084.0	21382667	6127633	0.29	3.96E-03	ALB	1.9536	6.1289	4.5055	1.27E-03	1.45E-02	-1.0035
			305	1	2	794.9	212540000	27026667	0.13	1.96E-03							
			298	1	2	863.9	216883333	26548667	0.12	1.44E-04							
			97	1	2	987.5	135626333	13136433	0.10	7.11E-03							
			257	1	2	847.0	87783000	6540100	0.07	1.14E-03							
			117	1	4	793.8	59130667	3887750	0.07	6.50E-03							
			160	1	3	940.8	575220000	28546333	0.05	1.68E-03							
			463	1	2	536.2	69895000	2682550	0.04	1.92E-02							
			402	1	3	696.4	148740000	5320100	0.04	1.20E-02							
			597	1	2	620.8	163196667	5693467	0.03	2.67E-03							
			229	1	2	619.3	284810000	9082167	0.03	8.56E-05							
			569	1	3	628.3	4536400										
			588	1	2	771.3	13804000										
			499	1	2	754.9	21198333										
			75	1	3	891.1	22158667										
			581	1	2	916.9	26848667										
			264	1	3	967.1	34013000										
			310	0.5	4	1080.5	42471667										
			558	1	2	654.4	68081667										
			383	1	3	854.0	68391333										
			219	1	2	630.8	104322667										
			214	1	3	520.9	166796667										
P04406	GAPDH	Glyceraldehyde-3-phosphate dehydrogenase	194	1	2	629.3	57157667	99222000	1.74	4.24E-03	GAPDH	1.0920	14.8930	6.9715	4.82E-05	2.43E-03	2.3707
			117	1	2	554.3	29715667	7332267	0.25	2.50E-02							
P01236	PRL	Prolactin	215	1	2	581.3	60229667	13244000	0.22	6.75E-05	PRL	-11.8908	12.9988	-7.0186	4.56E-05	2.37E-03	2.4268
			209	1	3	425.6	62347667	6615500	0.11	4.54E-03							
P06576	ATP5B	ATP synthase subunit beta, mitochondrial	522	1	2	564.8		40025000			ATP5B	1.7318	13.8762	7.3689	3.06E-05	1.99E-03	2.8353
P08865	RPSA	40S ribosomal protein SA	11	1	2	930.0	2031750				RPSA	-1.1474	12.2183	-5.7994	2.06E-04	5.28E-03	0.8765
P01242	GH2	Growth hormone variant	198	1	2	648.3	20045333				GH2	-11.4395	7.8750	-30.7801	6.85E-11	5.16E-07	13.8468
Q6ZN40	TPM1	Tropomyosin 1 (Alpha)	29	1	2	516.3	2542500				TPM1	2.2713	10.5676	7.2960	3.32E-05	2.05E-03	2.7515
P0DMV8	HSPA1A	Heat shock 70 kDa protein 1A	319	1	2	642.9	5140100				HSPA1A	-1.7989	11.7926	-4.7067	9.40E-04	1.22E-02	-0.6952
P05109	S100A8	Protein S100-A8	92	1	2	512.7	3562033				S100A8	4.4654	9.6276	4.6025	1.10E-03	1.33E-02	-0.8542
P04075	ALDOA	Fructose-bisphosphate aldolase A	330	1	2	568.3	9008850				ALDOA	1.2343	14.3796	5.4707	3.19E-04	6.64E-03	0.4222
			200	1	3	1073.5	12204667										
P07602	PSAP	Prosaposin	413	1	2	621.3	13188333	2534867	0.19	5.37E-03	PSAP	1.1000	11.9910	3.5359	5.78E-03	3.56E-02	-2.5605
			438	1	3	783.7	23948333										
P67936	TPM4	Tropomyosin alpha-4 chain	82	1	2	759.8	11726800				TPM4	-2.4119	9.3950	-3.9310	3.07E-03	2.42E-02	-1.9133
			11	1	2	621.3	20472333										
P14136	GFAP	Glial fibrillary acidic protein	260	1	2	654.3	6595600				GFAP	-4.2956	8.0496	-4.7014	9.48E-04	1.23E-02	-0.7033
P02647	APOA1	Apolipoprotein A-I	157	1	2	597.8	6918400	1258900	0.18	3.58E-03	APOA1	-1.8597	5.1153	-8.3617	1.06E-05	1.14E-03	3.9117
			262	1	2	778.9	6098750										
			230	1	2	728.9	8688850										
			206	1	2	600.3	10627500										
			130	1	2	711.9	18472967										
			164	1	3	656.3	59276000										
P56381	ATP5E		37	1	2	624.3	1963150	3165967	1.61	1.19E-02	ATP5E	1.8347	8.0226	4.8737	7.37E-04	1.06E-02	-0.4437
			21	1	2	619.3	7391700	2780333	0.38	2.11E-03							

LogFC >= 1: upregaled DEG. LogFC <= -1: downregulated DEGs.

**Table 3:** Cluster analysis of KEGG pathways, MFs, CCs, and BPs enriched with overlapped molecules (DAPs; invasive DEGs) in NF-PitNETs.

Cluster	Category	ID	Term	Count	%	P Value	Overlapped molecules (DAPs; invasive DEGs)
**Cluster 1**	Cellular components	GO:0043209	myelin sheath	6	23.08	1.41E-06	HSPA8, ATP5B, ATP5A1, ALB, SLC25A4, GFAP
	Biological process	GO:0006754	ATP biosynthetic process	4	15.38	9.15E-06	ATP5B, ATP5E, ATP5A1, ALDOA
	Molecular functions	GO:0016887	ATPase activity	5	19.23	1.41E-04	HSPA8, ATP5B, ATP5E, ATP5A1, HSPA1A
	Molecular functions	GO:0046933	proton-transporting ATP synthase activity, rotational mechanism	3	11.54	2.82E-04	ATP5B, ATP5E, ATP5A1
	Cellular components	GO:0005753	mitochondrial proton-transporting ATP synthase complex	3	11.54	3.44E-04	ATP5B, ATP5E, ATP5A1
	Biological process	GO:0042776	mitochondrial ATP synthesis coupled proton transport	3	11.54	4.04E-04	ATP5B, ATP5E, ATP5A1
	Molecular functions	GO:0046961	proton-transporting ATPase activity, rotational mechanism	3	11.54	6.70E-04	ATP5B, ATP5E, ATP5A1
	Molecular functions	GO:0022857	transmembrane transporter activity	3	11.54	2.37E-03	ATP5B, ATP5E, ATP5A1
	Pathway	hsa05012	Parkinson’s disease	4	15.38	6.55E-03	ATP5B, ATP5E, ATP5A1, SLC25A4
	Cellular components	GO:0005759	mitochondrial matrix	4	15.38	8.76E-03	ATP5B, MTHFD1L, ATP5E, ATP5A1
	Pathway	hsa05010	Alzheimer’s disease	4	15.38	1.04E-02	ATP5B, ATP5E, ATP5A1, GAPDH
	Pathway	hsa05016	Huntington’s disease	4	15.38	1.49E-02	ATP5B, ATP5E, ATP5A1, SLC25A4
	Cellular components	GO:0005739	mitochondrion	6	23.08	2.73E-02	ATP5B, MTHFD1L, ATP5A1, PSAP, SLC25A4, HSPA1A
	Pathway	hsa00190	Oxidative phosphorylation	3	11.54	5.12E-02	ATP5B, ATP5E, ATP5A1
**Cluster 2**	Molecular functions	GO:0016887	ATPase activity	5	19.23	1.41E-04	HSPA8, ATP5B, ATP5E, ATP5A1, HSPA1A
	Biological process	GO:0046034	ATP metabolic process	3	11.54	9.46E-04	HSPA8, ATP5B, HSPA1A
**Cluster 3**	Biological process	GO:0061621	canonical glycolysis	3	11.54	6.23E-04	TPI1, ALDOA, GAPDH
	Biological process	GO:0006096	glycolytic process	3	11.54	1.07E-03	TPI1, ALDOA, GAPDH
	Biological process	GO:0006094	gluconeogenesis	3	11.54	1.79E-03	TPI1, ALDOA, GAPDH
	Pathway	hsa00010	Glycolysis/Gluconeogenesis	3	11.54	1.44E-02	TPI1, ALDOA, GAPDH
	Pathway	hsa01230	Biosynthesis of amino acids	3	11.54	1.65E-02	TPI1, ALDOA, GAPDH
	Pathway	hsa01200	Carbon metabolism	3	11.54	3.81E-02	TPI1, ALDOA, GAPDH
**Cluster 4**	Cellular components	GO:0031012	extracellular matrix	4	15.38	6.67E-03	HSPA8, ATP5B, ATP5A1, GAPDH
	Cellular components	GO:0016020	membrane	8	30.77	2.00E-02	HSPA8, ATP5B, TPM4, MTHFD1L, ATP5A1, RPSA, ALDOA, GAPDH

### Confirmation of DAPs in NF-PitNETs

**Figure 9:**
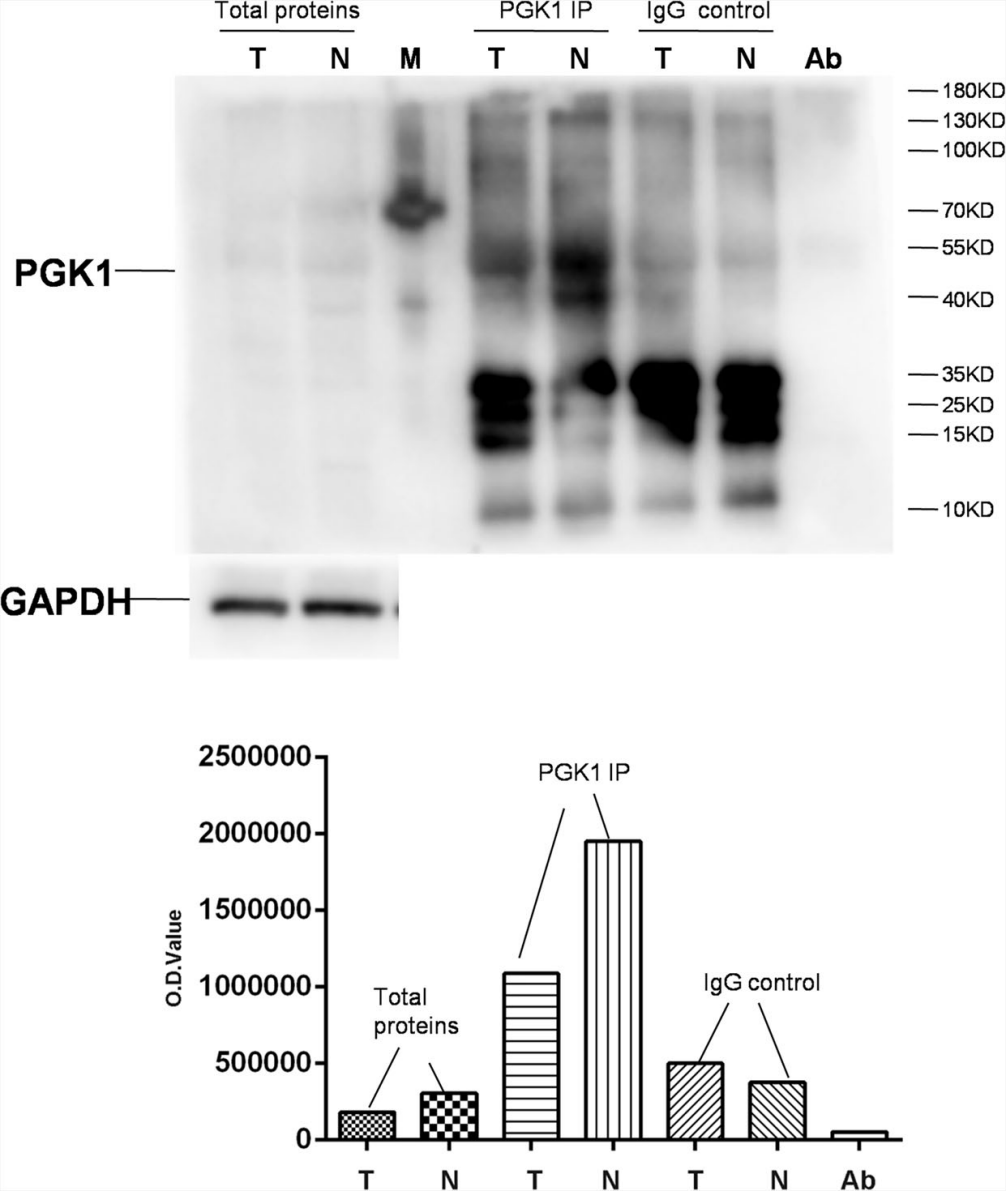
Semiquantitative analysis of acetylated PGK1 between NF-PitNETs and controls. PGK1 in protein samples extracted from NF-PitNET and control tissues was immunoprecipitated (IP) with anti-PGK1 antibody. A negative control immunoprecipitation experiment was performed with the normal mouse IgG antibody but not anti-PGK1 antibody to test the specificity of anti-PGK1 antibody. The IP products (PKG1 and IgG), anti-PGK1 antibodies (Ab), and total protein samples (tumor; control) were simultaneously immunoblotted with anti-acetyl-lysine antibody. T = NF-PitNETs. N = controls. M = markers.

A randomly selected DAP - PGK1 was used for further analysis with IP and western blot experiments (**Figure 9**). PGK1 was a down-acetylated protein in NF-PitNETs relative to controls identified with acetylomics. Acetylation at different lysine residues in PGK1 was able to positively or negatively regulate its enzymatic activities, which initiated or altered some signaling pathways, such as metabolism or autophagy, leading to tumor formation or progression (67–69). Acetylated PGK1 functioned in signaling pathways such as glycometabolism, carbon metabolism, and biosynthesis of amino acids. The decreased acetylation level of PGK1 in NF-PitNETs might trigger one switch, such as metabolic reprogramming, to induce pituitary tumorigenesis or NF-PitNET development. It emphasized the potential roles of the decreased acetylation level of PGK1 in the occurrence and development of NF-PitNETs.

## Discussion

This present study provided the first quantitative profiling of protein acetylation in NF-PitNET tissues. A total of 296 proteins with 517 acetylated sites was identified in NF-PitNETs compared to control pituitaries. The KEGG pathways, MFs, CCs, and BPs enriched with DAPs were clustered into 14 functional categories, which demonstrated that DAPs were widely involved in cellular biological processes and signaling pathways associated with metabolism, gene expression, cell adhesion, and immune system. Among 18 statistically significant KEGG signaling pathways enriched with DAPs, twelve pathways were metabolism-related pathways. Immunoprecipitation and western blotting analysis semi-quantitatively validated that acetyl-PGK1, a protein widely involved in glycometabolism, was decreased in NF-PitNETs. Furthermore, overlapping analysis of acetylomics and ubiquitinomics, and of DAP data and invasive DEGs data, found: (i) proteins that were modified by both acetyl and ubiquitin in NF-PitNETs were involved in nucleosome or ribosome, hemoglobin, prolactin, ubiquitin hydrolase, membrane proteins, and proteins constituting cytoskeleton; and acetylation levels of these proteins were decreased, whereas their ubiquitination levels were increased in NF-PitNETs. (ii) The invasiveness-related acetylated proteins were mainly involved in biological processes and signaling pathways about metabolism and energy yield, which suggested that NF-PitNET invasive behaviors might be acetylation-mediated metabolic reprogramming process.

Many tumor cells prefer to provide bioenergetics and growth requirements through glycolysis, rather than oxidative phosphorylation, during tumor growth progression, even with sufficient oxygen and normal mitochondria. Because glycolysis is able to provide sufficient cellular ATPs, and additional important metabolites to support the biosynthetic demands of consecutive cell proliferation (70). It is recognized that PitNETs also displayed very low levels of oxygen consumption, which was similar with other malignant tumors (71). A recent study found that PitNETs presented lactate progressive accumulation in cells, which suggests a bioenergetic metabolic shift from aerobic oxidation towards glycolysis metabolism to make tumor cells adapt to different energy requirements and enhance their survival chances (72). In NF-PitNETs, the vast majority of acetylation levels of lysine residues in proteins were decreased in glycolysis pathway, but increased in aerobic oxidation-related pathways, including TCA cycle and oxidative phosphorylation. This opposite acetylation status presented between glycolysis pathway and aerobic oxidation-related pathways indicated that the altered protein acetylation levels might involve in NF-PitNET metabolic reprogram through changing enzyme activity, stability, or other potential ways to affect NF-PitNET progression. This study found that PGK1 acetylation status was decreased in NF-PitNETs with LC/MS analysis, which was further semi-quantitatively validated with IP in combination with an acetyl-lysine immunoblot. PGK1 functions in glycolysis metabolism, which reversibly catalyzes 1,3-diphosphoglycerate to 3-phosphoglycerate, and subsequently transfers a phosphoryl group to ADP, and yields a molecule of ATP. Study found that PGK1 acetylation affected brain tumorigenesis through mediating autophagy and the increased acetylation level of PGK1, which was correlated with the poor prognosis in glioblastoma (73). The PGK1 acetylation was also found to promote its enzymatic activity and liver cancer cell metabolism, and significantly associate with poor prognosis of liver cancer patients (68). Thus, the decreased acetylation level of PGK1 in NF-PitNETs might also affect NF-PitNET tumorigenesis and progression through metabolic reprogramming, autophagy or other underlying mechanisms, but of which detail is needed to be further studied.

The invasive characteristics of NF-PitNETs have been a hot pot for a long time. Invasive PitNETs tend to suffer tumor postoperative residual and re-growth because of cavernous sinus invasion or the internal carotid artery encircling, which is able to cause poor prognosis. However, their underlying invasive mechanism remains unclear (74). This study found 26 molecules were differentially acetylated, and were invasiveness-related DEGs, which were obtained through comparative analysis of DAP data and invasive DEG data. Most of these overlapped molecules (DAPs; Invasive DEGs) mainly functioned in metabolism-associated biological processes and pathways. Thus, it seems reasonable to propose that acetylation-mediated metabolic reprogramming is associated with NF-PitNET invasive characteristics. Previous study found that metabolic stress, such as oxidative stress or hypoxia, was able to enhance invasiveness, angiogenesis, stemness, and metastatic potential of tumor cells (75). According to previous study, aerobic glycolysis was considered as one metabolic reprogram paradigm that might occur in NF-PitNETs (71, 72). It acidified extracellular matrix (ECM), and subsequently activated matrix metalloproteinase and cathepsin to increase ECM degradation, which paved the way for many basic cell behaviors, including cell migration (76). Some overlapped molecules, such as HSPA8 and GAPDH, were found to be regulated by acetylation and deacetylation and associated with invasiveness of cancers (77–81). The interesting mechanisms that how protein acetylation affects NF-PitNETs metabolic reprogram to enhance its invasiveness are worthy further investigating, which is promised to provide a novel therapeutic target for NF-PitNET radical treatment.

The co-regulation of acetylation and ubiquitination at specific lysine sites in some proteins might affect tumor development, such as Lys382 of p53 and Lys125 of SRSF5 (23, 25). One of mechanisms of acetylation and ubiquitination co-regulation is the direct competition between acetyl and ubiquitin at the same lysine sites to control protein stability. Under the complex of histone deacetylases and E3 (a factor transferring ubiquitin to proteins) actions, the substrate protein lysine sites relieve acetyl and are subsequently ubiquitinated to be degraded by the proteasome. The complex of histone acetyltransferases and ubiquitin-specific proteases in contrast would be free these ubiquitinated lysine residues and acetylated lysine residues, which protected the target proteins from proteasome-mediated protein degradation and maintained their stability (82). This study found that lysines co-regulated by acetylation and ubiquitination were down-acetylated but up-ubiquitinated in NF-PitNETs, which indicated that acetyl and ubiquitin directly competed for the same lysine, which might result in proteasome-mediated degradation of these proteins, and affect NF-PitNET development.

Moreover, NF-PitNET and control tissue samples were very limited and precious, only very limited amount of proteins were obtained for subsequent quantitative acetylomics analysis. More acetylated sites and acetylated proteins are promised to be identified when the increased NF-PitNET protein samples available in future acetylomics analysis. This acetylome map of human NF-PitNETs described in this study is one component in the long-term program to find out NF-PitNET-specific acetylated proteins to clarify molecular mechanisms of NF-PitNETs. To achieve this goal, quantitative acetylomics needs to be further developed in the future.

In summary, the current study provided the first human acetylomic data in NF-PitNETs, offered a valuable resource for further study in NF-PitNET tumorigenesis and progression, which contributed to the discovery of effective biomarkers for early diagnosis and therapy of NF-PitNETs.

## Conclusion

This study provided a comprehensive approach that integrated anti-acetyl antibody-based enrichment, LC-MS/MS, and literature-based bioinformatics to discover *in vivo* acetylated proteins and their acetylation sites, and to rationalize the functions of DAPs. A total of 296 acetylated proteins with 517 acetylated lysine sites provided a quantitative status of lysine acetylation in NF-PitNETs, and their bioinformatics analysis provided a new insight into the roles of protein lysine acetylation in formation and development of NF-PitNETs. The acetylation levels of more than half acetylated proteins were decreased in NF-PitNETs. Acetylation-mediated metabolic reprogramming might be considered as one of the underlying mechanisms in tumorigenesis and invasiveness of NF-PitNETs. Further investigation is needed to ascertain the biological significance of these lysine acetylation events and their relevance to NF-PitNET pathogenesis.

## Data Availability Statement

The datasets presented in this study can be found in online repositories. The names of the repository/repositories and accession number(s) can be found in the article/**Supplementary Material**.

## Ethics Statement

The studies involving human participants were reviewed and approved by the Xiangya Hospital Medical Ethics Committee of Central South University; the University of Tennessee Health Science Center Internal Review Board. The patients/participants provided their written informed consent to participate in this study.

## Author Contributions

SW analyzed data, carried out western blot experiment and immunoprecipitation experiment, prepared figures and tables, designed and wrote the manuscript. JL, JY, BL, and NL participated in partial data analysis and experiments. XZ conceived the concept, designed experiments and manuscript, instructed experiments, analyzed data, obtained the acetylomics data, supervised results, coordinated, wrote and critically revised manuscript, and was responsible for its financial supports and the corresponding works. All authors contributed to the article and approved the submitted version.

## Funding

This work was supported by the Shandong First Medical University Talent Introduction Funds (to XZ), the Hunan Provincial Hundred Talent Plan (to XZ), Shandong Provincial Natural Science Foundation (ZR202103020356 to XZ), the National Natural Science Foundation of China (82172866), and the Academic Promotion Program of Shandong First Medical University (2019ZL002).

## Conflict of Interest

The authors declare that the research was conducted in the absence of any commercial or financial relationships that could be construed as a potential conflict of interest.

## Publisher’s Note

All claims expressed in this article are solely those of the authors and do not necessarily represent those of their affiliated organizations, or those of the publisher, the editors and the reviewers. Any product that may be evaluated in this article, or claim that may be made by its manufacturer, is not guaranteed or endorsed by the publisher.

## Abbreviations

BPs, biological processes; CCs, cellular components; DAPs, differentially acetylated proteins; DEGs, differentially expressed proteins; DTT, dithiothreitol; F-PitNET, functional pituitary neuroendocrine tumor; GEO, Gene Expression Omnibus; GO, gene ontology; HEPEs, 2-hydroxyethyl; HPLC, high performance liquid chromatography; IAP, immunoaffinity purification; IP, immunoprecipitation; KATs, lysine acetyltransferases; KDACs, lysine deacetylases; LC, liquid chromatography; MFs, molecular functions; MS, mass spectrometry; MS/MS, tandem mass spectrometry; NF-PitNET, nonfunctional pituitary neuroendocrine tumor; PitNETs, pituitary neuroendocrine tumors; PGK1, phosphoglycerate kinase 1; PTM, post-translational modification; S/N, signal-to-noise; TFA, trifluoroacetic acid.
