# Supplementary material for: Quantitative Acetylomics Revealed Acetylation-Mediated Molecular Pathway Network Changes in Human Nonfunctional Pituitary Neuroendocrine Tumors
Source: Front Endocrinol (Lausanne). 2021 Oct 12;12:753606. doi: 10.3389/fendo.2021.753606 (PMC8546192; doi:10.3389/fendo.2021.753606)
Supplement: Supplementary file 2 [file Presentation_1.pptx]

## Slide 1
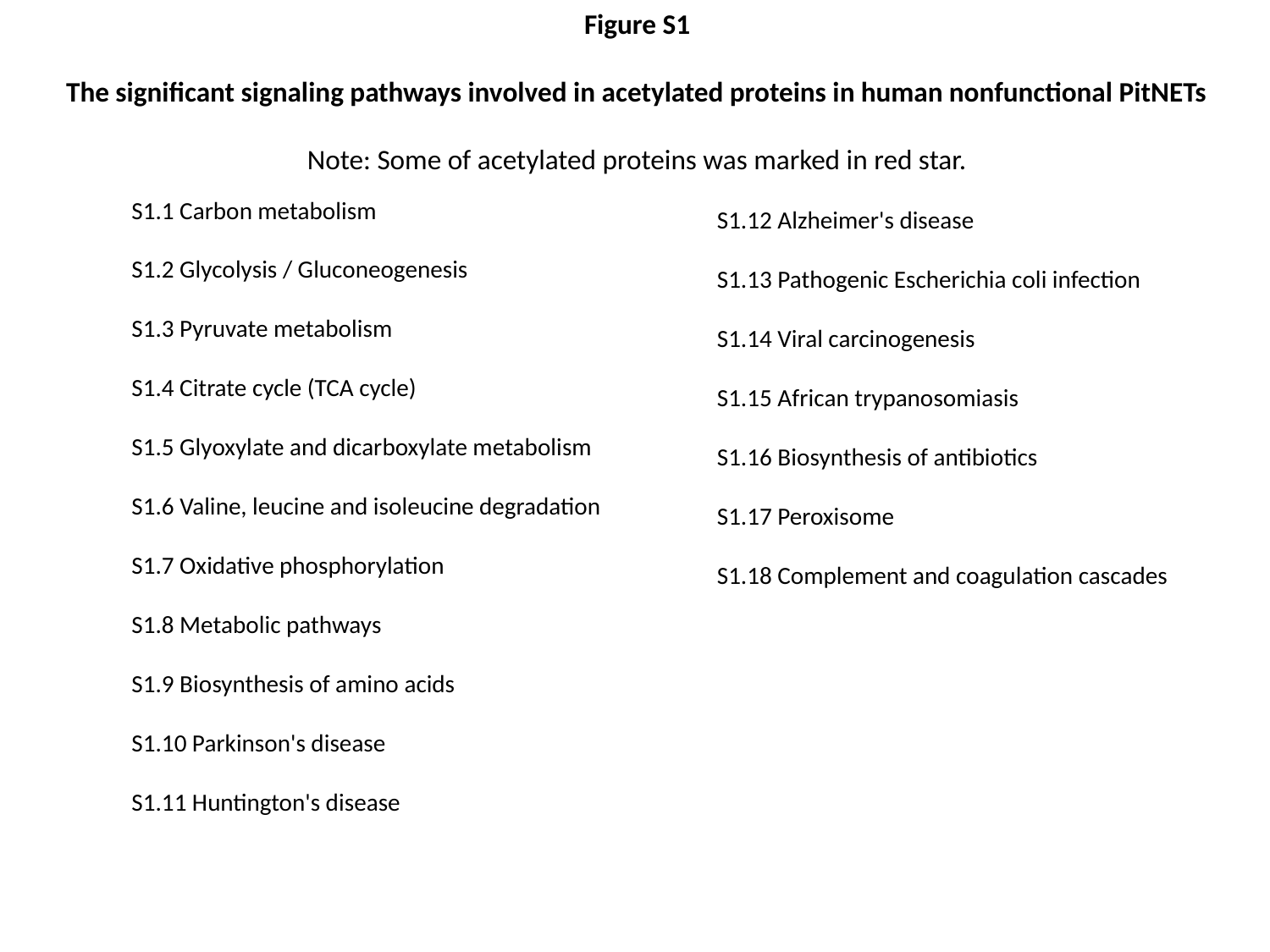

Figure S1
The significant signaling pathways involved in acetylated proteins in human nonfunctional PitNETs
Note: Some of acetylated proteins was marked in red star.
S1.1 Carbon metabolism
S1.2 Glycolysis / Gluconeogenesis
S1.3 Pyruvate metabolism
S1.4 Citrate cycle (TCA cycle)
S1.5 Glyoxylate and dicarboxylate metabolism
S1.6 Valine, leucine and isoleucine degradation
S1.7 Oxidative phosphorylation
S1.8 Metabolic pathways
S1.9 Biosynthesis of amino acids
S1.10 Parkinson's disease
S1.11 Huntington's disease
S1.12 Alzheimer's disease
S1.13 Pathogenic Escherichia coli infection
S1.14 Viral carcinogenesis
S1.15 African trypanosomiasis
S1.16 Biosynthesis of antibiotics
S1.17 Peroxisome
S1.18 Complement and coagulation cascades

## Slide 2
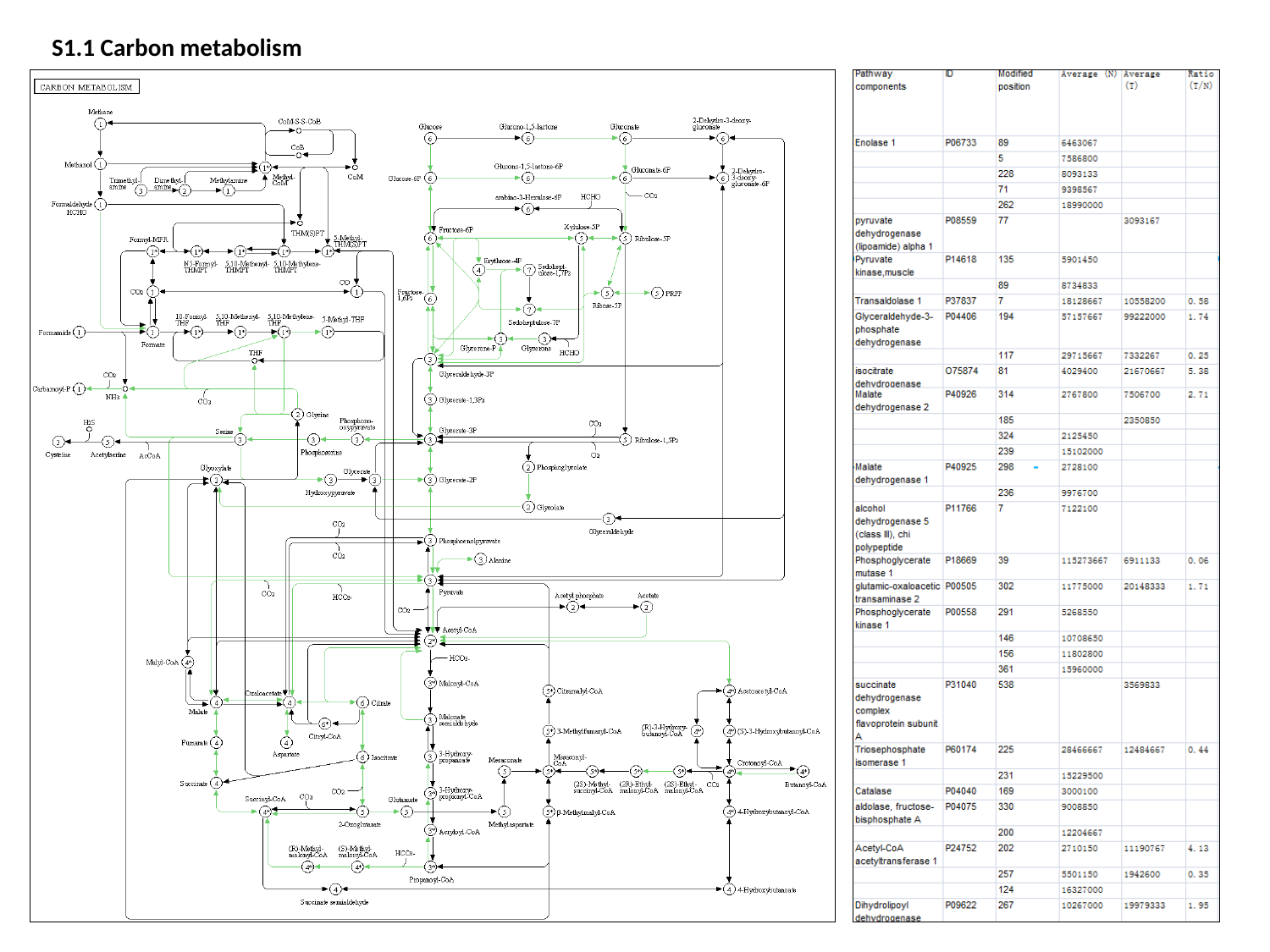

S1.1 Carbon metabolism

## Slide 3
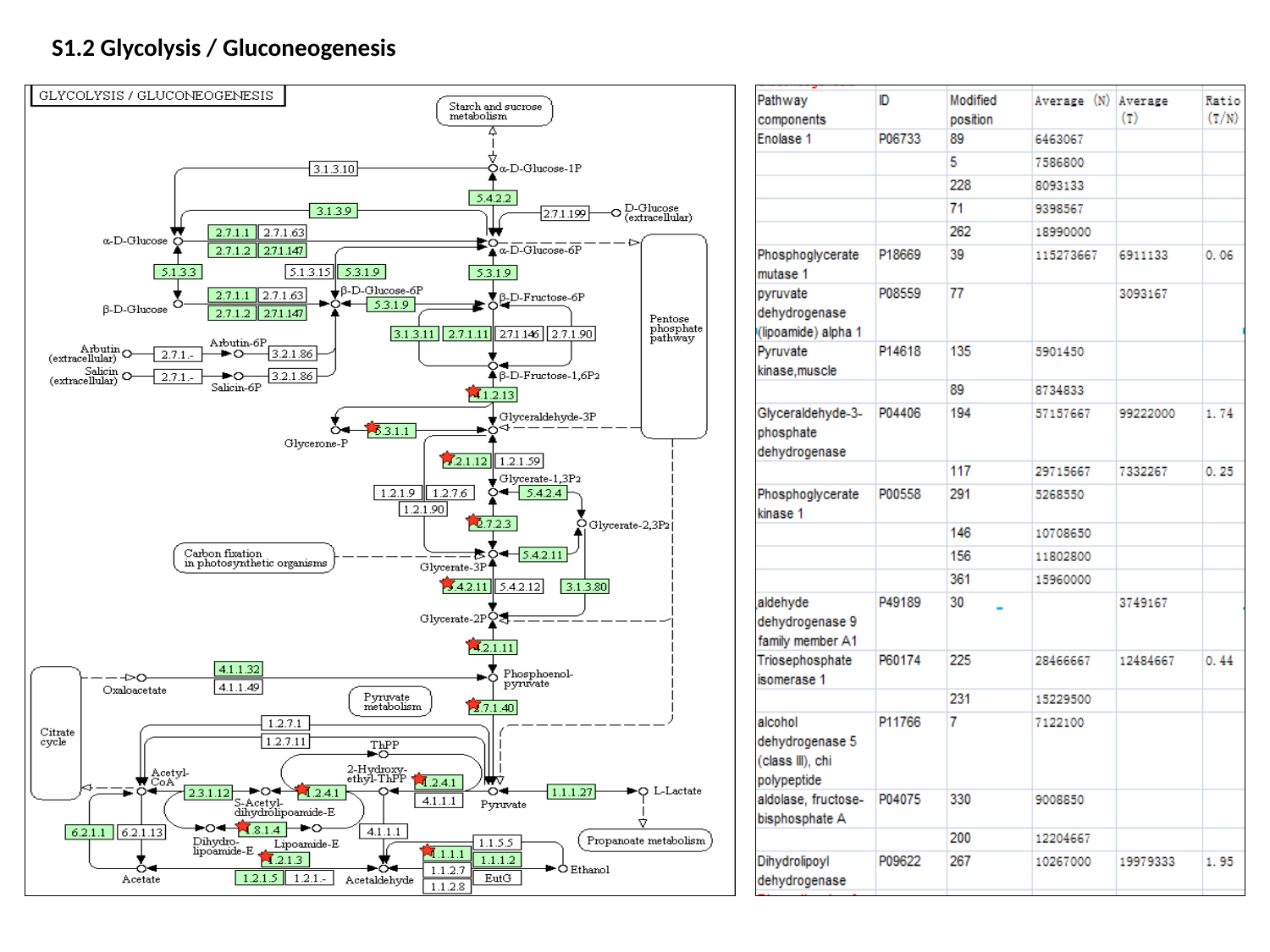

S1.2 Glycolysis / Gluconeogenesis

## Slide 4
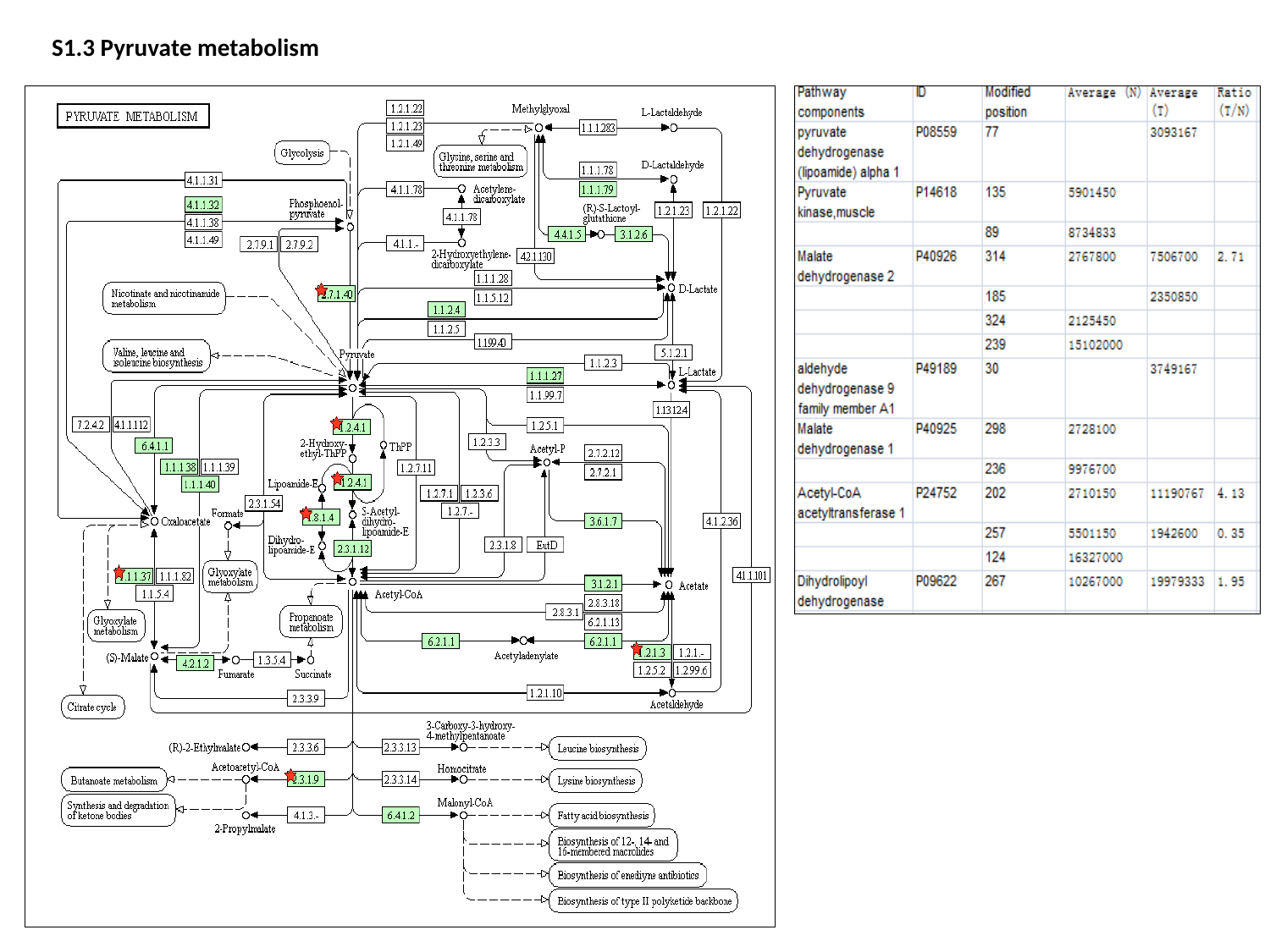

S1.3 Pyruvate metabolism

## Slide 5
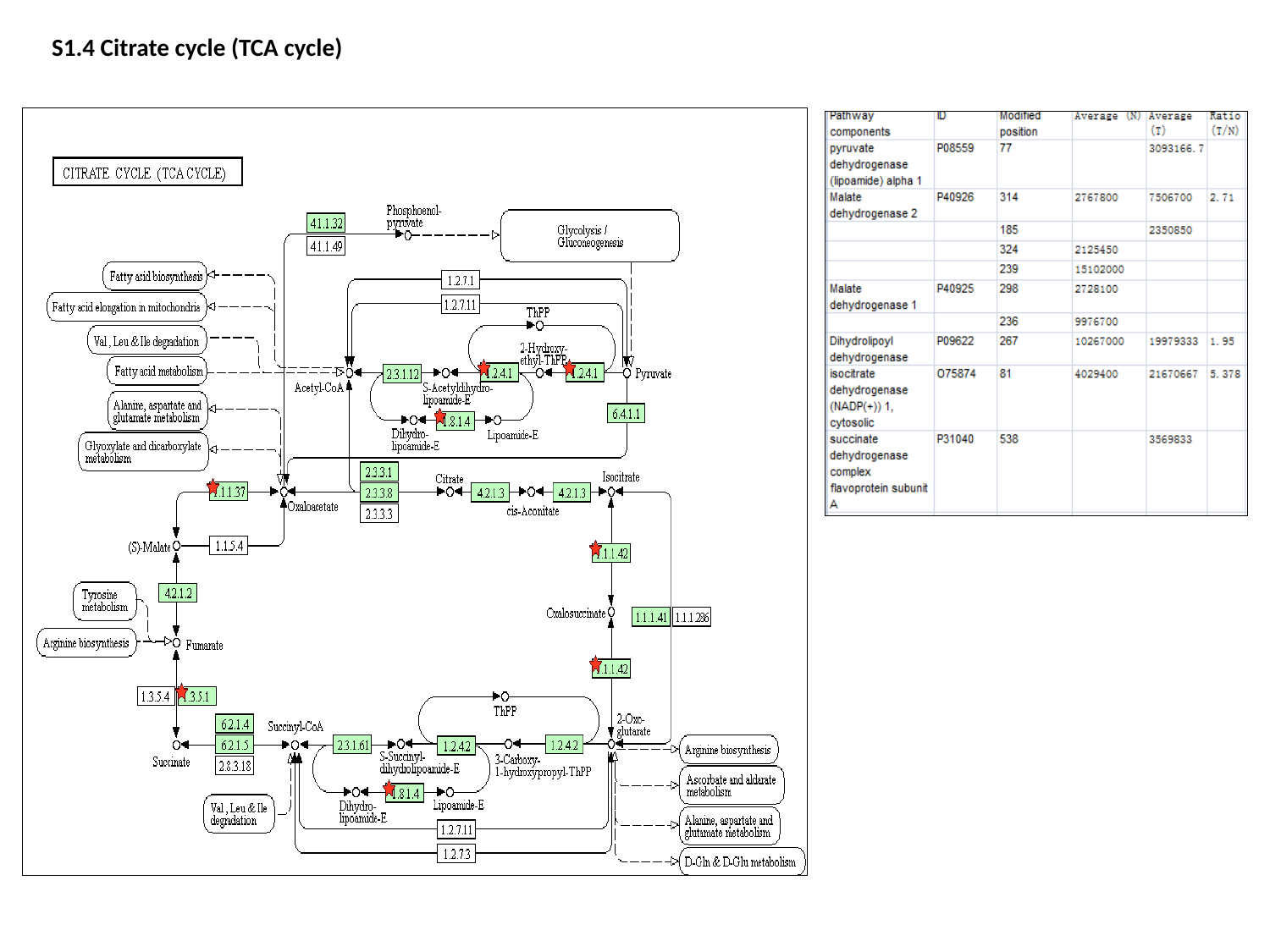

S1.4 Citrate cycle (TCA cycle)

## Slide 6
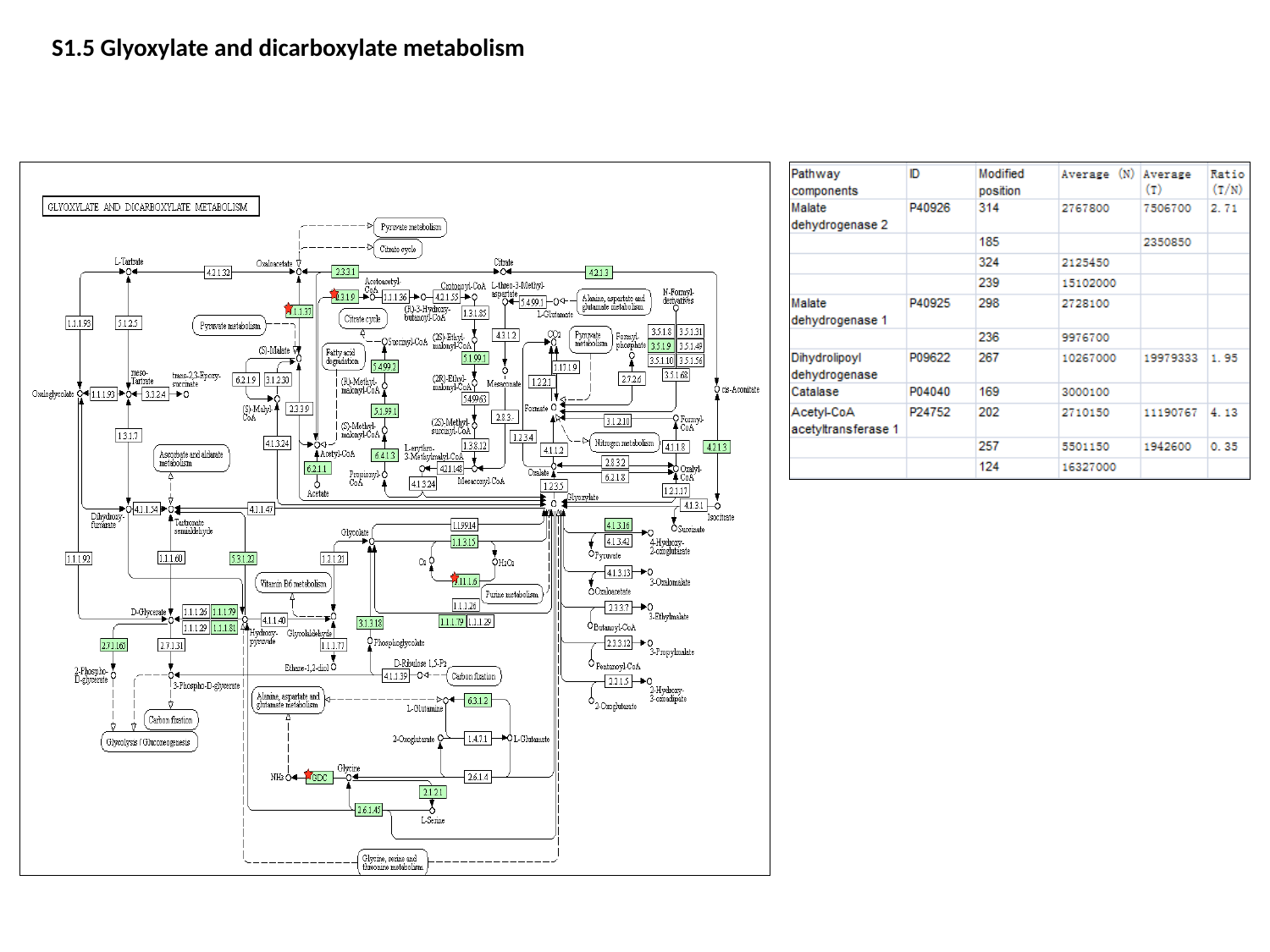

S1.5 Glyoxylate and dicarboxylate metabolism

## Slide 7
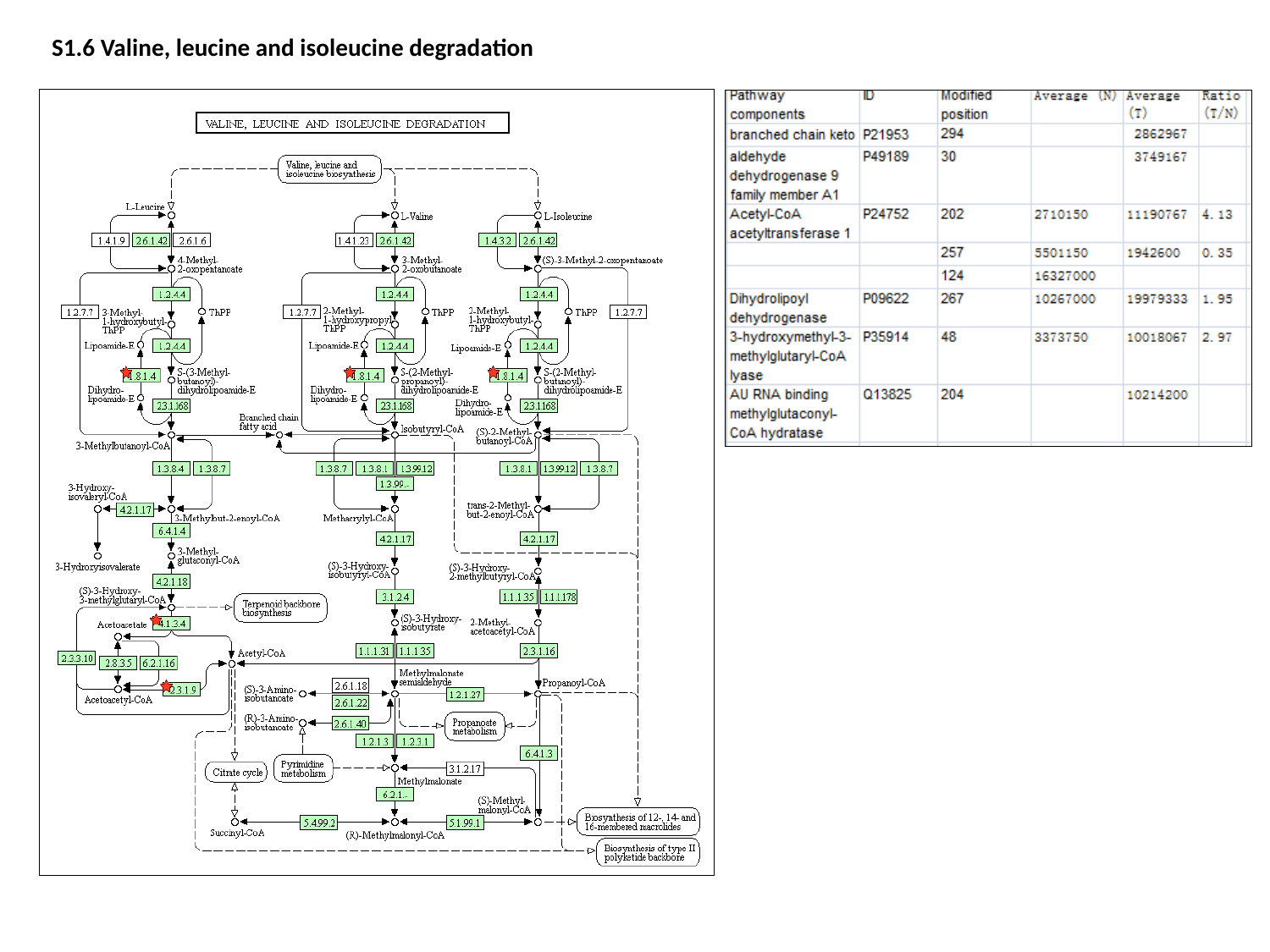

S1.6 Valine, leucine and isoleucine degradation

## Slide 8
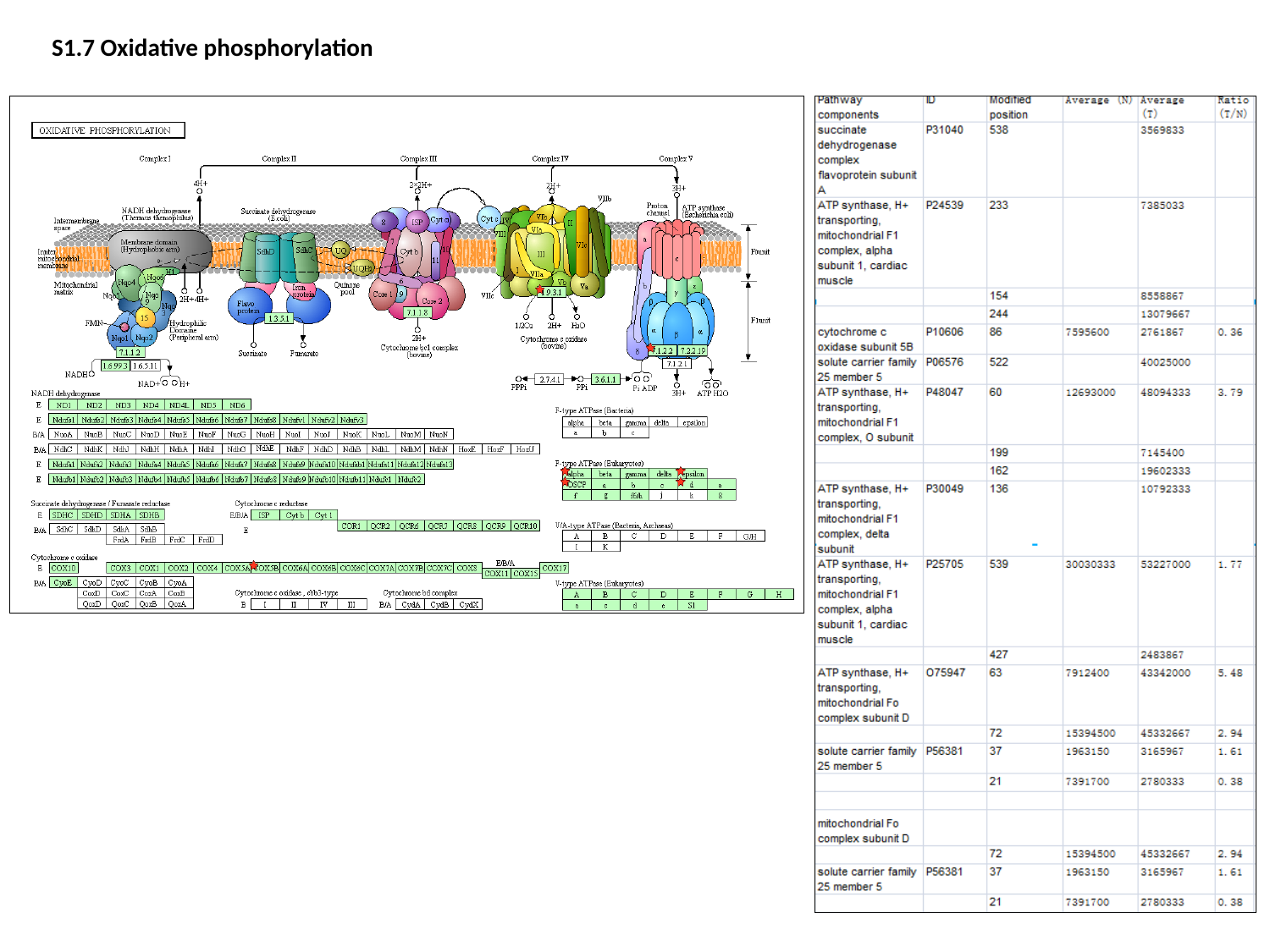

S1.7 Oxidative phosphorylation

## Slide 9
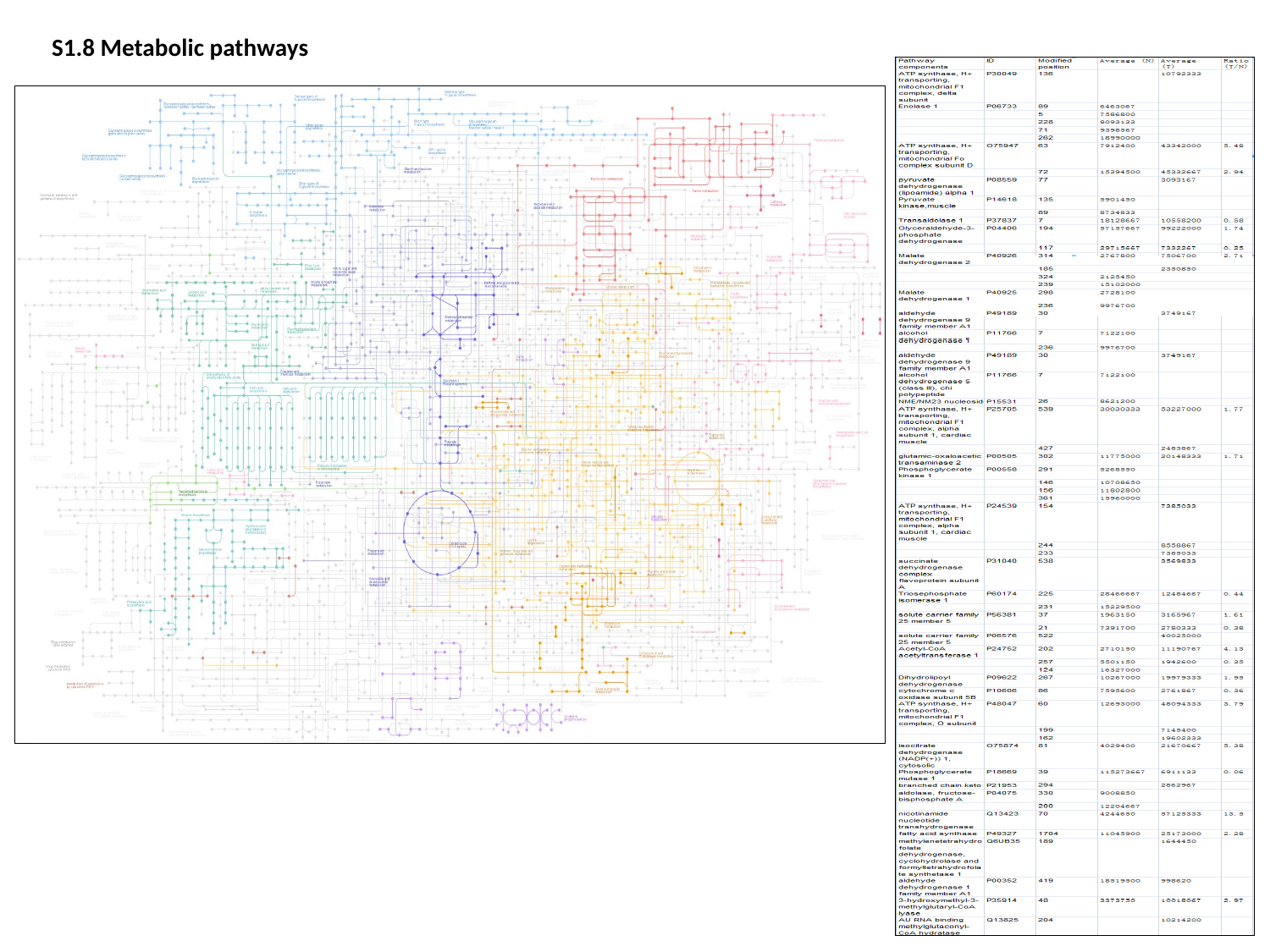

S1.8 Metabolic pathways

## Slide 10
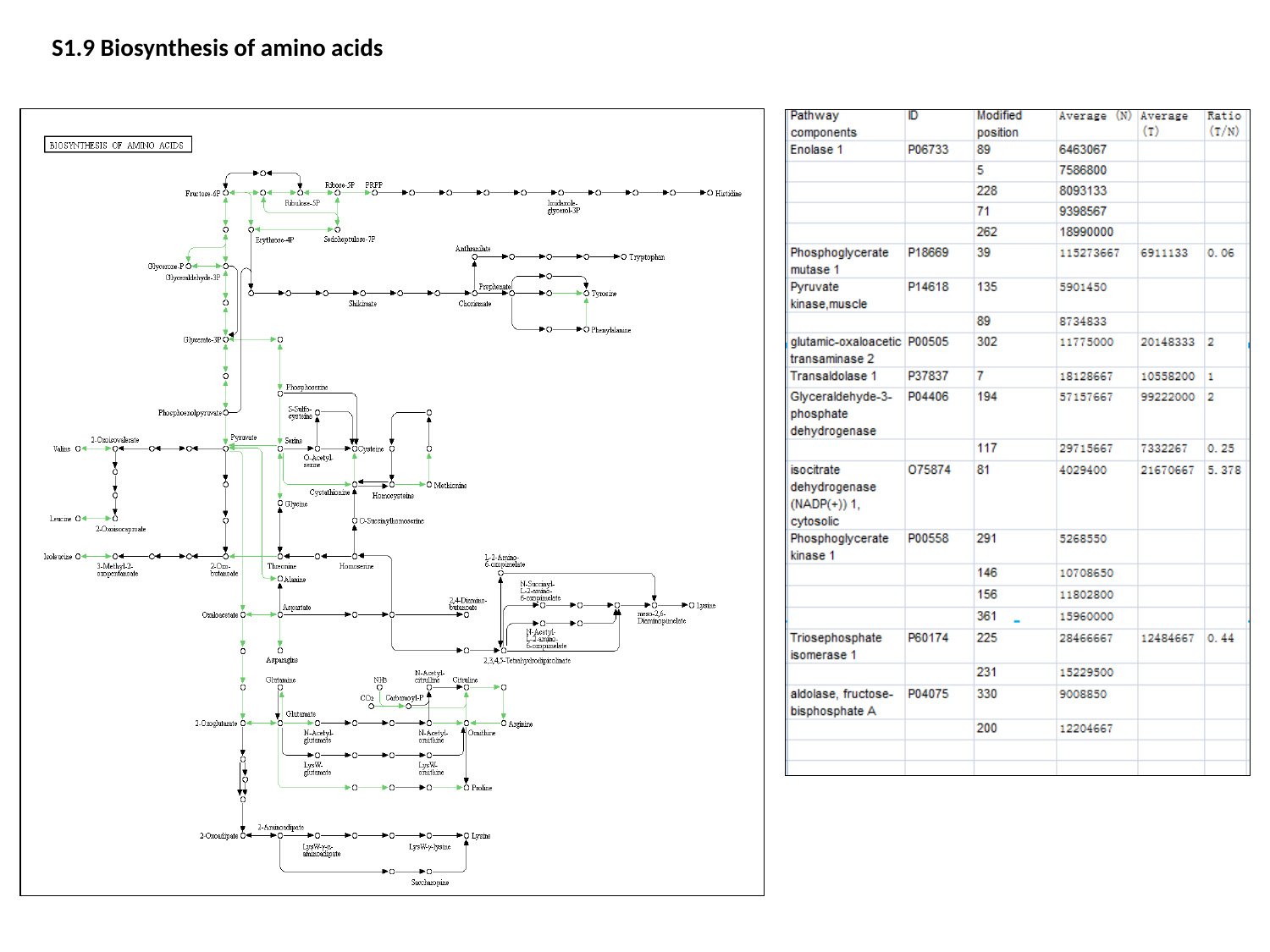

S1.9 Biosynthesis of amino acids

## Slide 11
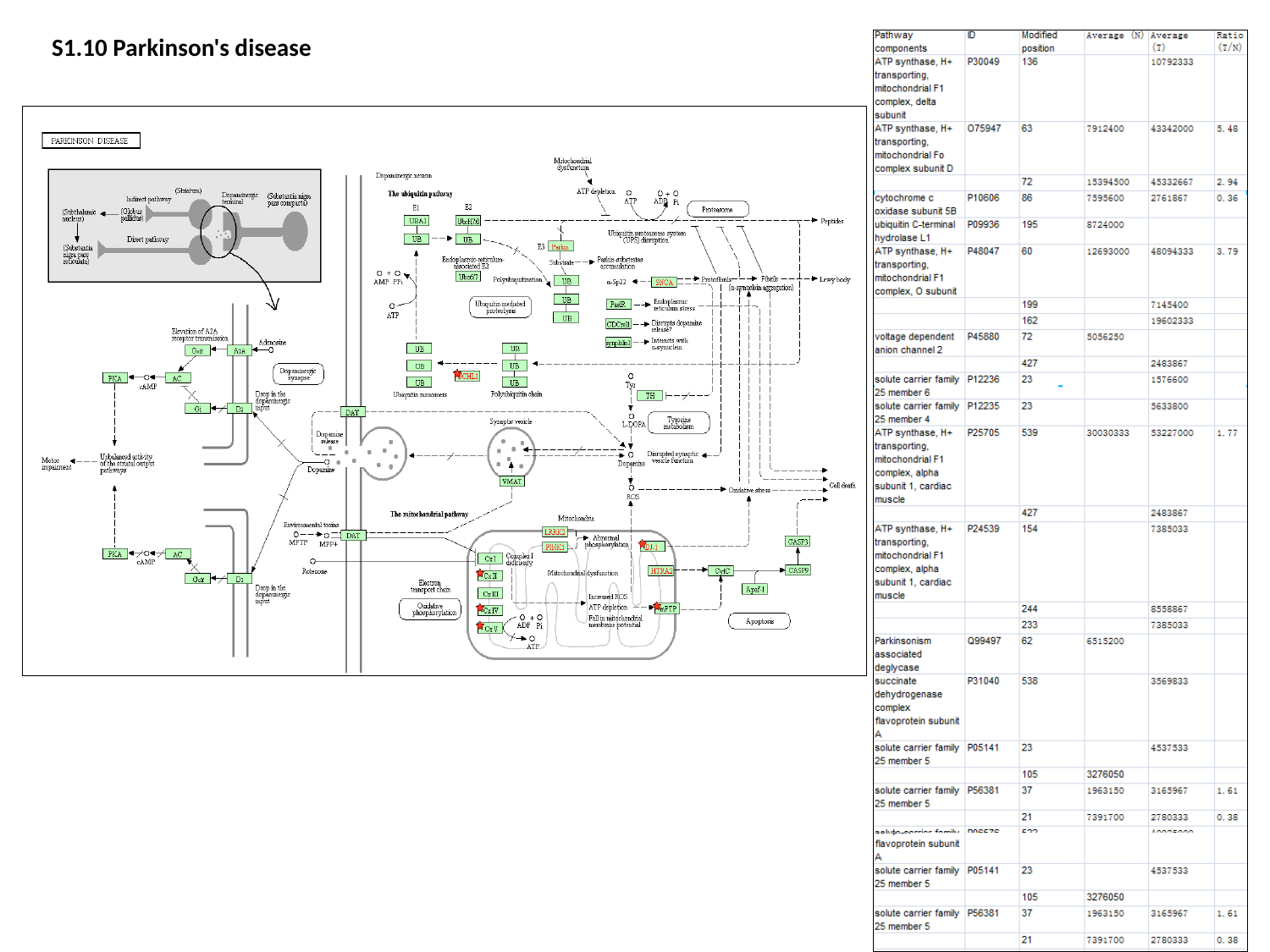

S1.10 Parkinson's disease

## Slide 12
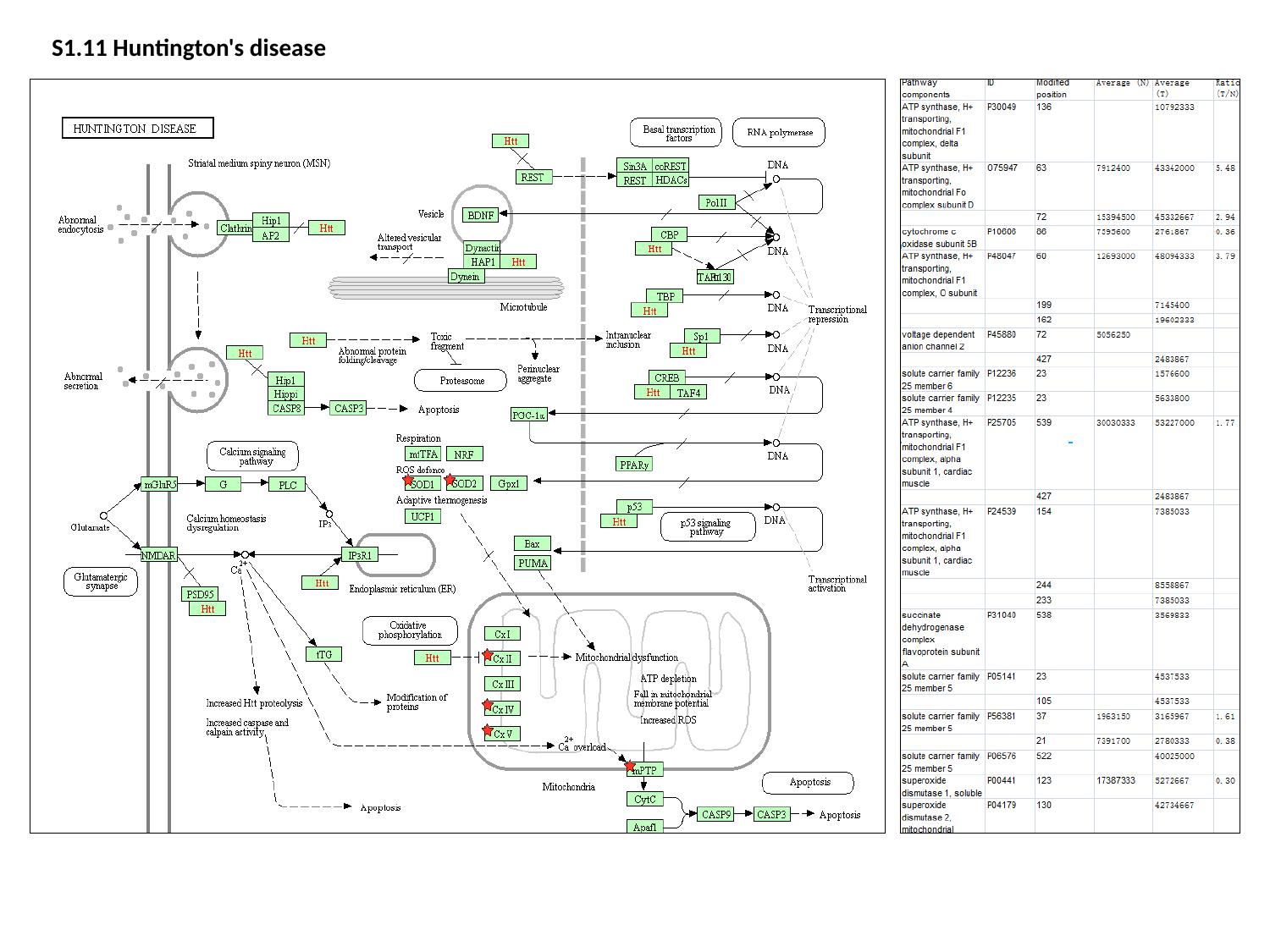

S1.11 Huntington's disease

## Slide 13
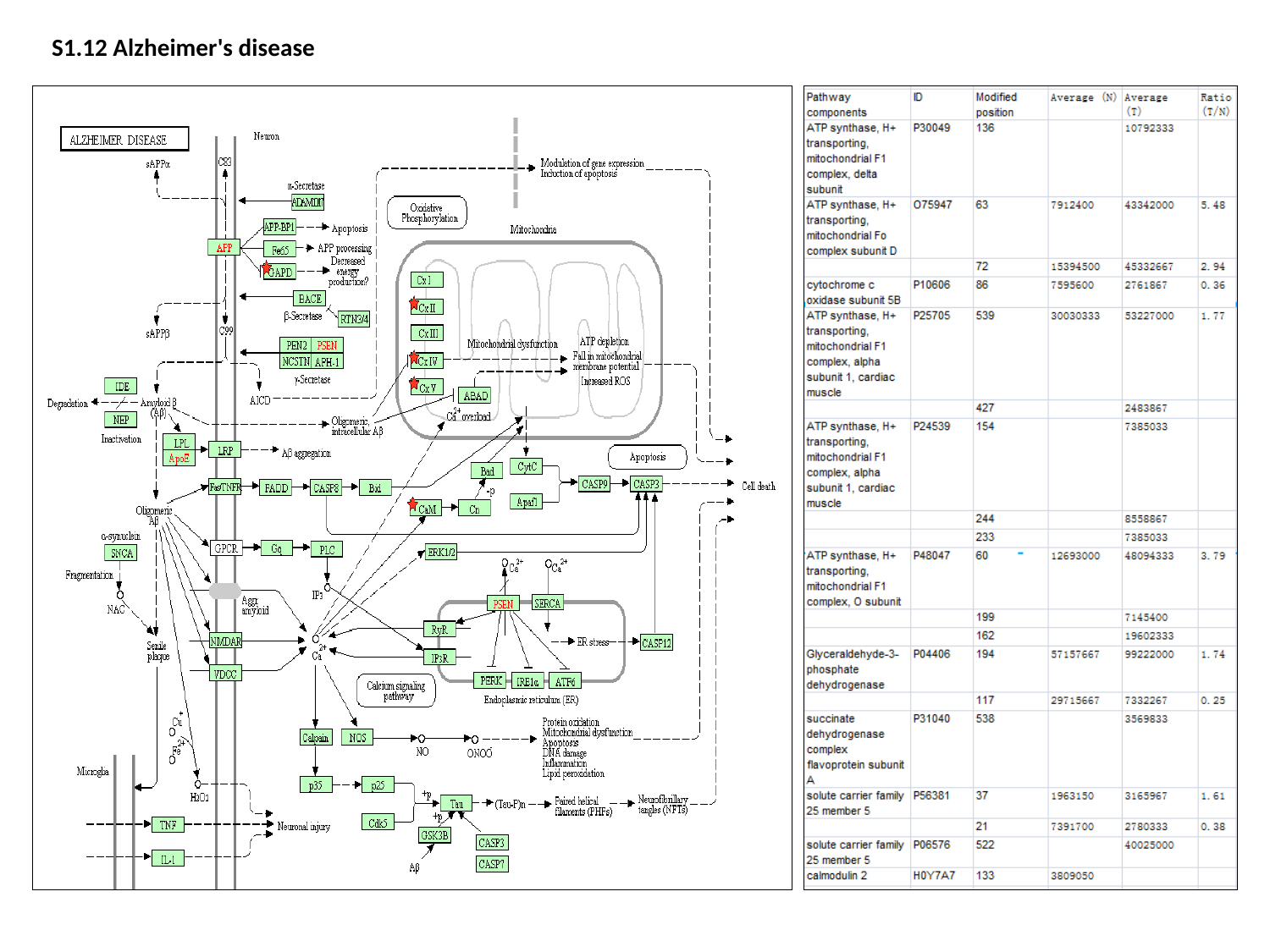

S1.12 Alzheimer's disease

## Slide 14
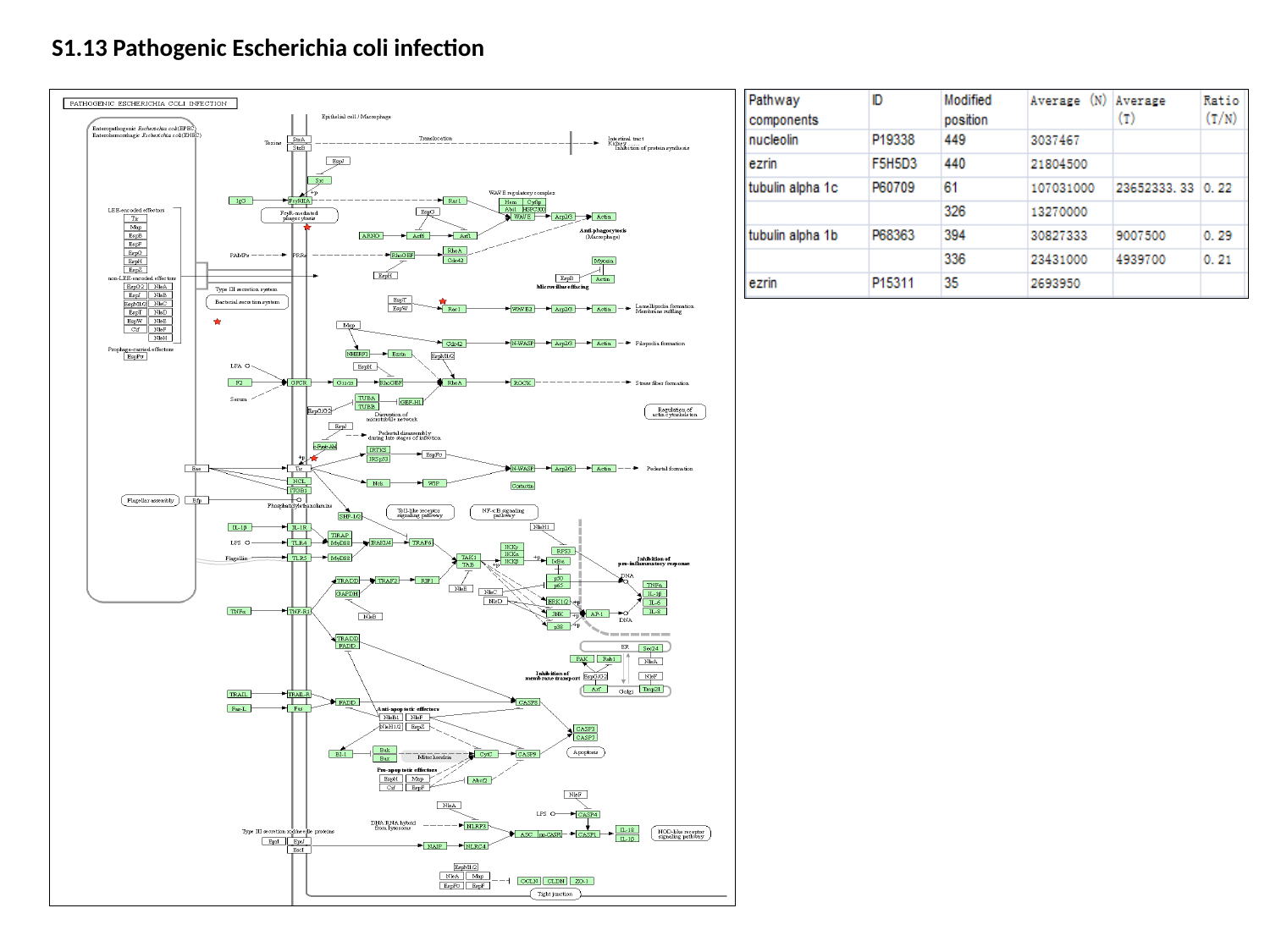

S1.13 Pathogenic Escherichia coli infection

## Slide 15
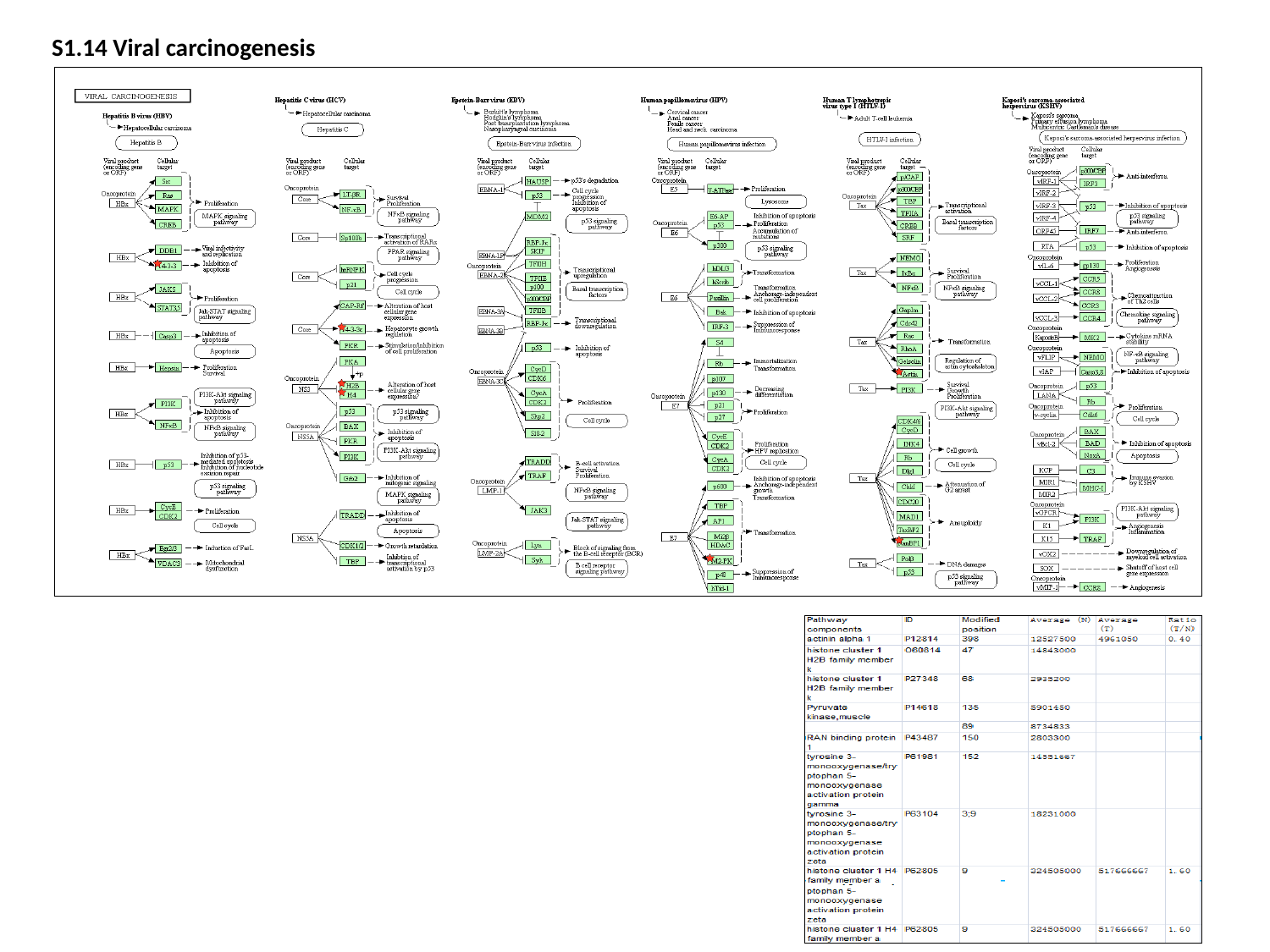

S1.14 Viral carcinogenesis

## Slide 16
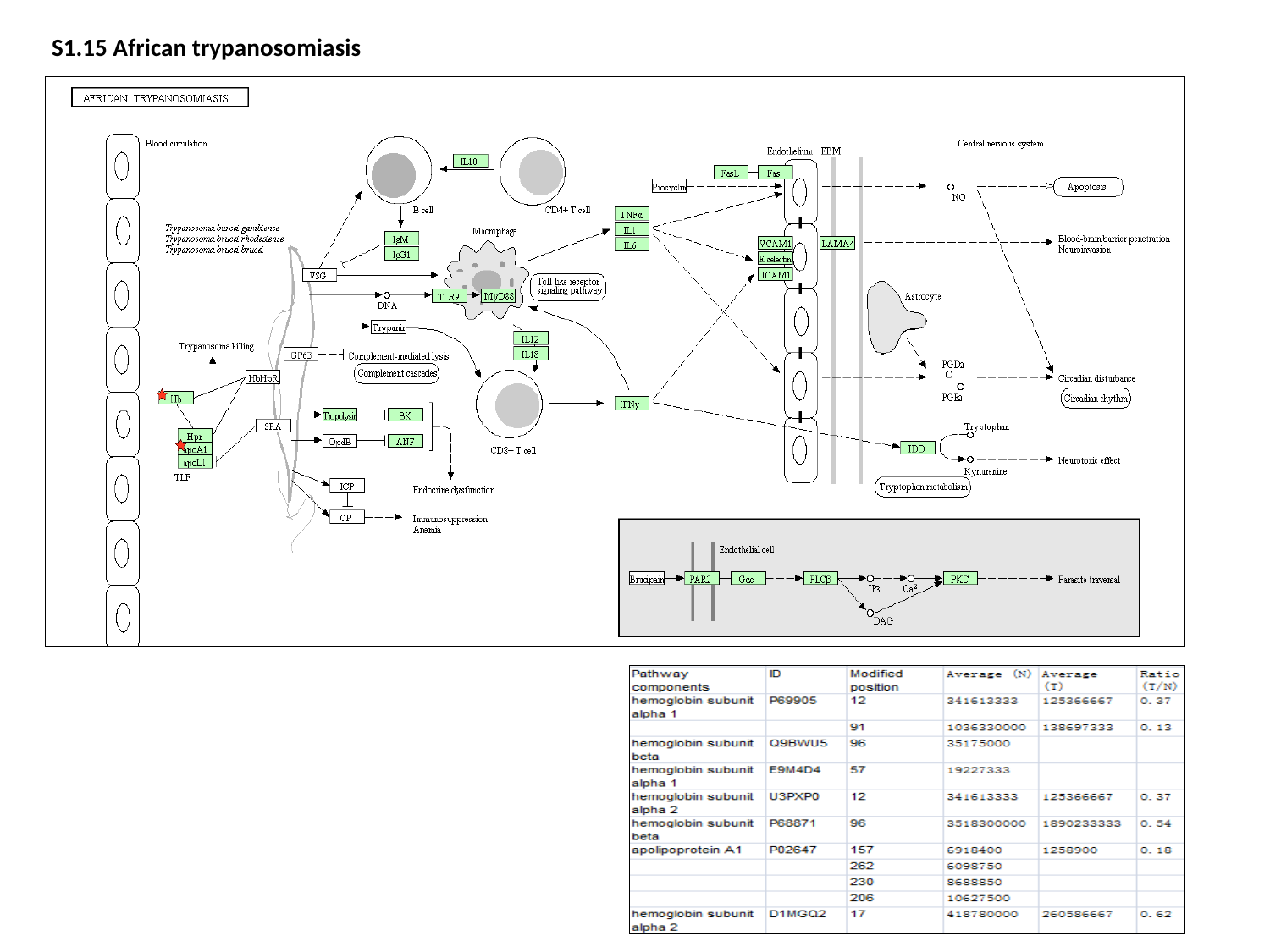

S1.15 African trypanosomiasis

## Slide 17
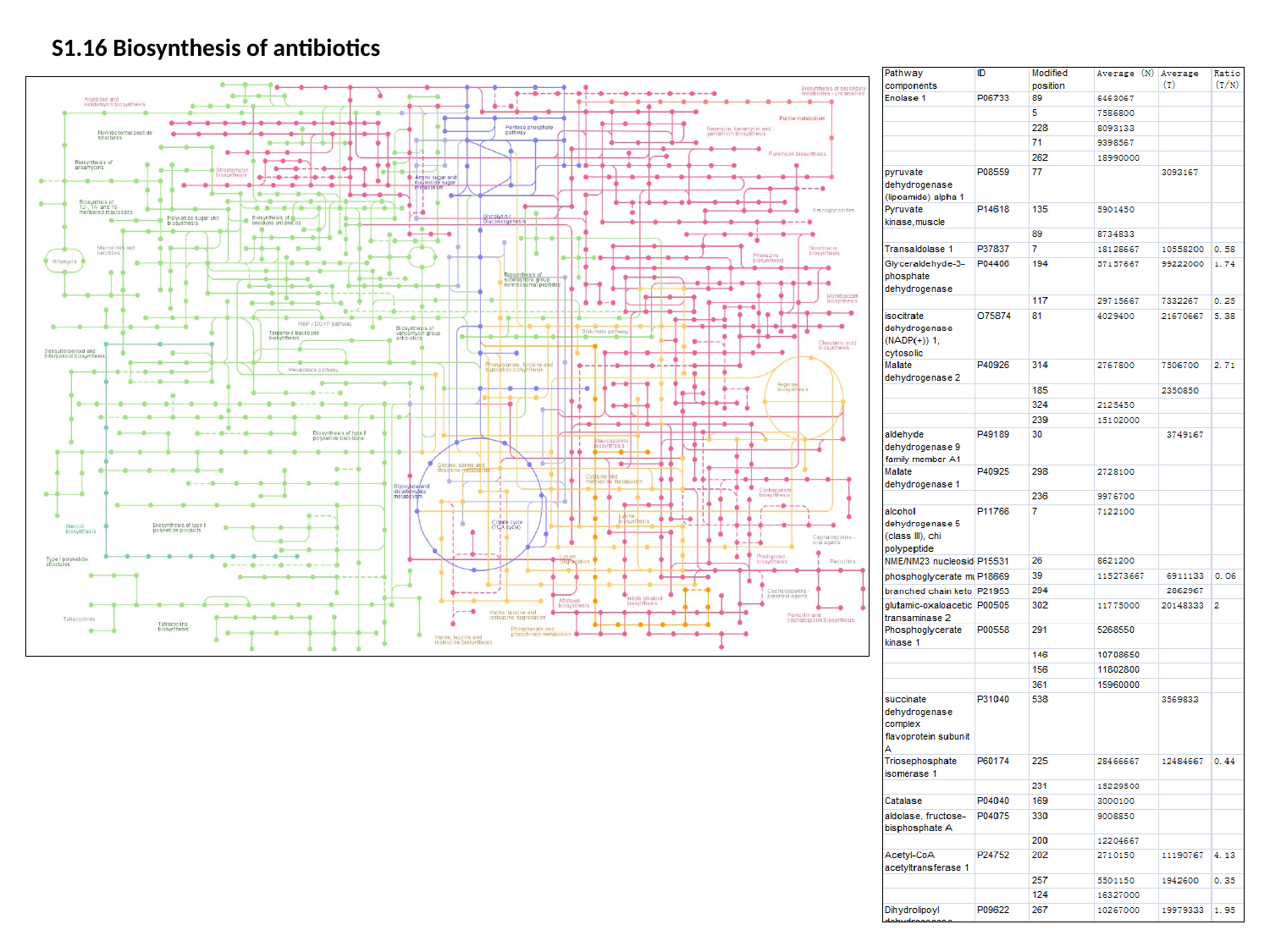

S1.16 Biosynthesis of antibiotics

## Slide 18
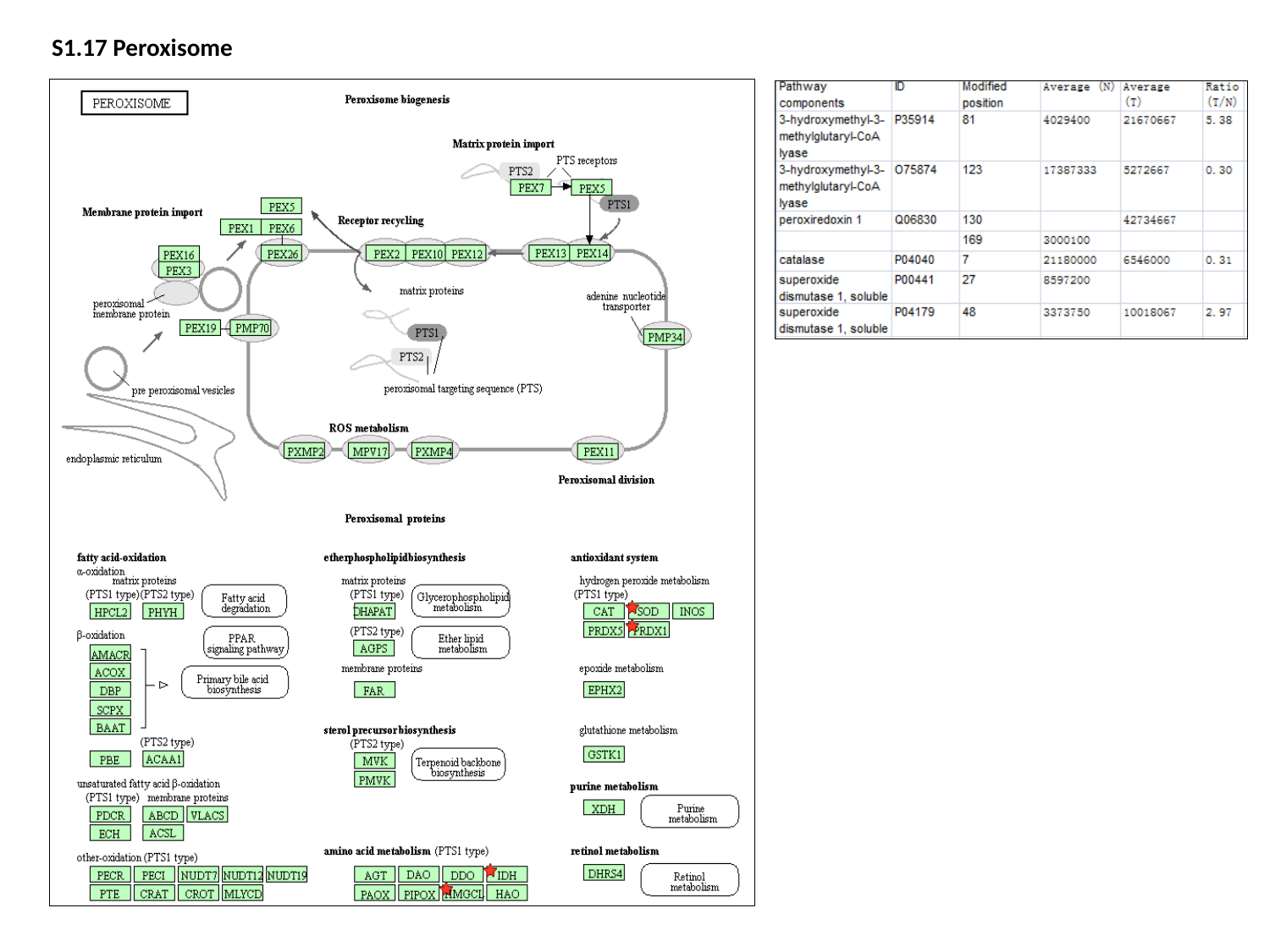

S1.17 Peroxisome

## Slide 19
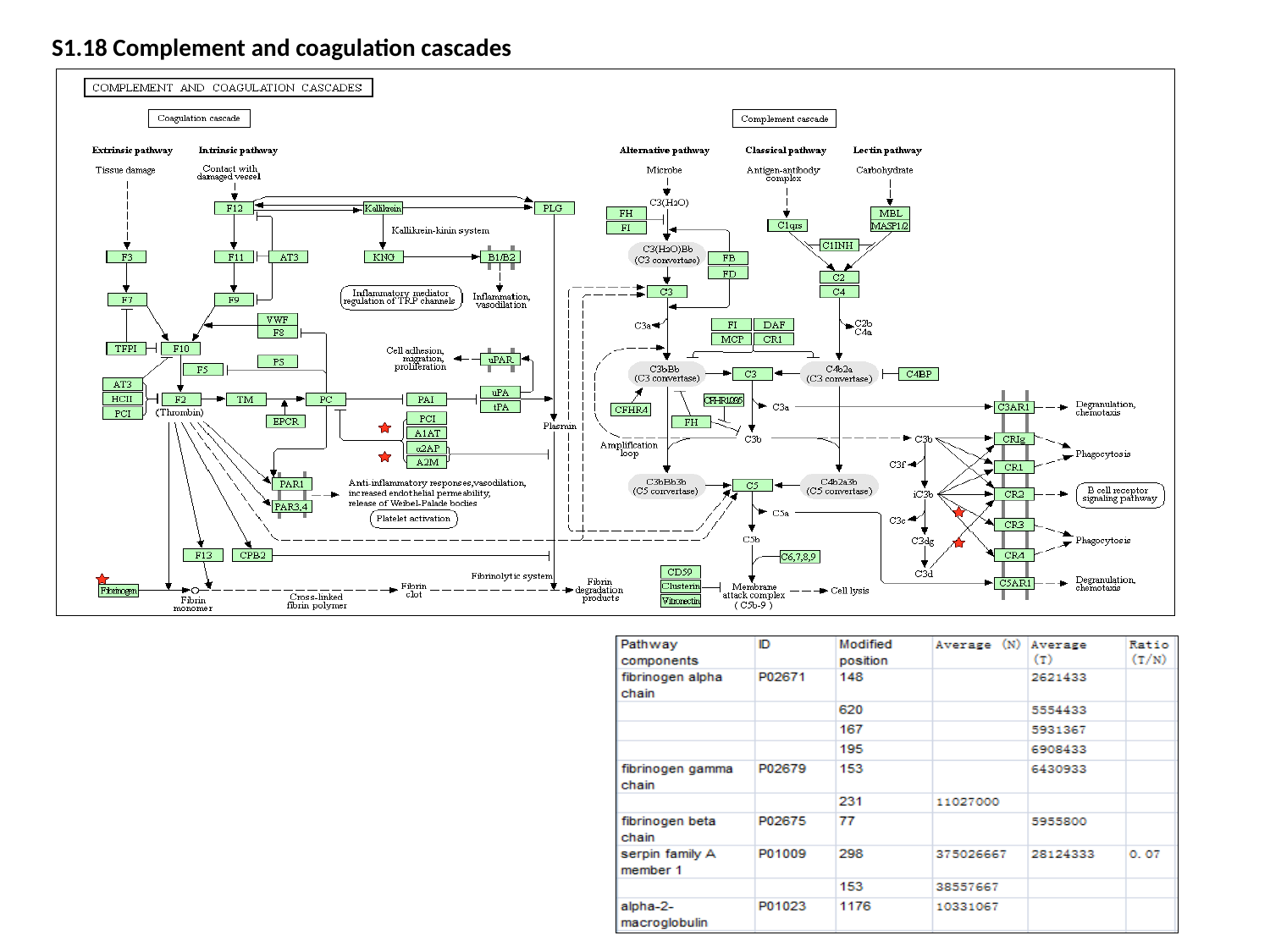

S1.18 Complement and coagulation cascades
