# Supplementary material for: Quantitative Acetylomics Revealed Acetylation-Mediated Molecular Pathway Network Changes in Human Nonfunctional Pituitary Neuroendocrine Tumors
Source: Front Endocrinol (Lausanne). 2021 Oct 12;12:753606. doi: 10.3389/fendo.2021.753606 (PMC8546192; doi:10.3389/fendo.2021.753606)
Supplement: Supplementary file 1 [file DataSheet_1.zip › Supplementary Table 1_v1.doc]

**Supplementary Table 1: Clinical information of NFPA and control tissue samples. DNT = do not test. ACTH = adrenocorticotropic hormone. GH = growth hormone. PRL = prolactin. FSH = follicle-stimulating hormone. LH =luteinizing hormone. TSH= thyroid-stimulating hormone. hGH = human growth hormone. NFPA = nonfunctional pituitary adenoma. IP = immunoprecipitation. WB = Western blot.**

| **Group** | **Sex** | **Age** | **Clinical information** | **Immunohistochemistry** | **Experiments** |
| --- | --- | --- | --- | --- | --- |
| NFPA | Male | 58 | NFPA in sellar region. Sellar floor bone destruction, enriched blood supply in tumor, and tumor size: 4.5 x 3 x 3 cm^3^ | ACTH (-), hGH (-), PRL (-), FSH (-), LH (-), TSH (-) | Proteomics |
|  | Male | 53 | NFPA in sellar region. Sellar floor bone thinning, and tumor size: 3 x 3 x 2.5 cm^3^ | ACTH (-), hGH (-), PRL (-), FSH (-), LH (-), TSH (-) | Proteomics |
|  | Female | 43 | NFPA in sellar region. Sellar floor bone thinning, enriched blood supply in tumor, and tumor size: 4 x 3 x 3 cm^3^ | ACTH (-), hGH (-), PRL (-), FSH (+), LH (-), TSH (-) | Proteomics |
|  | Female | 43 | NFPA in sellar region. Adehesion of surrounding tissues, and tumor size: 4.5 x 4 x6 cm^3^ | ACTH (-), hGH (-), PRL (-), FSH (+), LH (-), TSH (-) | Proteomics |
|  | Female | 51 | NFPA in sellar region. Adhesion of surrounding tissues, and tumor size: 4 x 4 x3.5 cm^3^ | ACTH (+), hGH (-), PRL (-), FSH (+), LH (-), TSH (-) | IP/WB |
|  | Female | 43 | NFPA in sellar region. Sellar floor bone thinning, enriched blood supply in tumor, and tumor size: 3.5 x 3 x 2.5 cm^3^ | ACTH (-), hGH (-), PRL (-), FSH (-), LH (-), TSH (-) | IP/WB |
|  | Female | 44 | NFPA in sellar region. Sellar floor bone thinning, and tumor size: 2.5 x 2.9 x 1.8 cm^3^ | ACTH (-), hGH (-), PRL (-), FSH (-), LH (-), TSH (-) | IP/WB |
| Control | Male | 36 | White, Multiple toxic materials. Blood alcohol = 0.5 g/L. Blood: HepB (+), HepC (−), HIV (−). | DNT | Proteomics; IP/WB |
|  | Female |  | White, 15 h gunshot wound to head. No drugs or alcohol. Blood: HepB (−), HepC (−), HIV (−). | DNT | Proteomics; IP/WB |
|  | Female | 34 | Black, Gunshot wound to chest. Blood alcohol = 0.3 g/L; no drugs. Blood: HepB (+), HepC (−), HIV (−). | DNT | Proteomics; IP/WB |
|  | Female | 40 | White, Multiple toxic compounds. Blood: HepB (+), HepC (+), HIV(−). | DNT | Proteomics; IP/WB |
|  | Male | 45 | White, Drowning. Blood alcohol = 3.1 g/L; no other drugs detected. Blood: HepB (+), HepC (+), HIV(−). | DNT | IP/WB |
